# Mapping the spreading routes of lymphatic metastases in human colorectal cancer

**DOI:** 10.1038/s41467-020-15886-6

**Published:** 2020-04-24

**Authors:** Chong Zhang, Lin Zhang, Tianlei Xu, Ruidong Xue, Liang Yu, Yuelu Zhu, Yunlong Wu, Qingqing Zhang, Dongdong Li, Shuohao Shen, Dongfeng Tan, Fan Bai, Haizeng Zhang

**Affiliations:** 1 Biomedical Pioneering Innovation Center (BIOPIC) and Translational Cancer Research Center, School of Life Sciences, First Hospital, Peking University, Beijing, 100871 China; 2 Department of Colorectal Surgery, National Cancer Center/National Clinical Research Center for Cancer/Cancer Hospital, Chinese Academy of Medical Sciences and Peking Union Medical College, Beijing, 100021 China; 3 State Key Lab of Molecular Oncology, National Cancer Center/National Clinical Research Center for Cancer/Cancer Hospital, Chinese Academy of Medical Sciences and Peking Union Medical College, Beijing, 100021 China; 4 Department of Pathology, National Cancer Center/National Clinical Research Center for Cancer/Cancer Hospital, Chinese Academy of Medical Sciences and Peking Union Medical College, Beijing, 100021 China; 5 Department of Pathology and GI Medical Oncology, The University of Texas M. D. Anderson Cancer Center, Houston, 77030 USA

**Keywords:** Cancer genomics, Gastrointestinal cancer, Metastasis

## Abstract

Lymphatic metastases are closely associated with tumor relapse and reduced survival in colorectal cancer (CRC). How tumor cells disseminate within the lymphatic network remains largely unknown. Here, we analyze the subclonal structure of 94 tumor samples, covering the primary tumors, lymph node metastases (LNMs), and liver metastases from 10 CRC patients. We portray a high-resolution lymphatic metastatic map for CRC by dividing LNMs into paracolic, intermediate, and central subgroups. Among the 61 metastatic routes identified, 38 (62.3%) are initiated from the primary tumors, 22 (36.1%) from LNMs, and 1 from liver metastasis (1.6%). In 5 patients, we find 6 LNMs that reseed 2 or more LNMs. We summarize 3 diverse modes of metastasis in CRC and show that skip spreading of tumor cells within the lymphatic network is common. Our study sheds light on the complicated metastatic pattern in CRC and has great clinical implications.

## Abstract (web summary)

Lymphatic metastases are closely associated with tumor relapse and reduced survival in colorectal cancer (CRC). Here, the authors analysed the primary tumours, lymph node metastasis and liver metastasis of ten CRC patients and reveal co-existence of diverse modes of metastasis in the same patient.

## Introduction

Colorectal cancer (CRC) is the second leading cause of cancer-related deaths worldwide^1,2^. Currently, effective treatment for CRC is challenging, since patients exhibit heterogeneous clinical outcomes and drug responses due to inter-tumor^3–7^ as well as intratumor heterogeneity^8–10^. Lymphatic and distant metastases are closely associated with reduced survival in CRC^1,2^. The manner in which these metastases are seeded is intensely debated^11^. In the prevalent sequential progression model, tumor cells first spread to lymph nodes and then disseminate to distant organs via the lymphatic system. Current CRC management guidelines are based on this model, in which lymph node metastasis (LNM) serves as an important prognostic factor and triggers lymphadenectomy to prevent tumor cells from further spreading to distant organs^12^.

However, a recent study using indel mutations in hypermutable, noncoding polyguanine repeats challenged this model by showing that lymphatic and distant metastases had distinct clonal origins in 65% (11/17) of CRC patients^13^. These results suggest that tumor cells in the primary tumor can directly spread to distant organs without traveling through the lymph nodes, thus questioning the necessity of lymphadenectomy to prevent disease progression. Nevertheless, this methodology was inadequate, since it queried a very limited genomic space and could not resolve the subclonal composition of these variants. Furthermore, most current studies focus on the comparison between LNMs and distant metastases but how tumor cells disseminate within the lymphatic network is less explored, and whether all LNMs have the same metastatic potential remains largely unknown. In addition, to determine whether LNMs contribute to the seeding of distant metastases, it is important to harvest as many clinically visible LNMs as possible; however, previous studies have involved <50% of detected LNMs^13–15^. Therefore, a more comprehensive analysis of metastatic routes in CRC is needed.

From an evolutionary perspective, a tumor is comprised of multiple subclones competing for growth and survival^16,17^. Tracing subclones from the primary tumor to metastases has enabled the reconstruction of subclonal structures and metastatic histories of multiple cancer types^18–20^ including prostate^21^, breast^22,23^, lung^24^, renal^25^, liver^26^, ovary^27^, urothelium^28^, and metastases in the brain^29^. In the present study, we seek to systematically investigate the lymphatic metastasis routes in CRC. We collect 55 LNMs with 28 matched multi-region samples from primary tumors in 10 CRC patients. In 3 of these patients, 11 multi-region samples from liver metastases (LMs) are also collected. High-quality exome sequencing data are retrieved from these samples and analyzed. Subsequently, we reconstruct the subclonal structure and infer the metastatic history for these cases. Both monoclonal and multiclonal seeding patterns are identified. We summarize the metastatic routes, identify three distinct modes of metastasis, and show that skip spreading of tumor cells is common among the metastasis network. We find that LNMs can have different metastatic potentials, with certain LNMs reseeding more than two metastases. Interestingly, we identify an LM-to-LM seeding, and also find that primary-to-LM and LNM-to-LM can coexist in the same patient. Our results reveal the detailed metastatic process of CRC and provide important clinical implications for CRC management.

## Results

### Patient cohort and research strategy

**Fig. 1:**
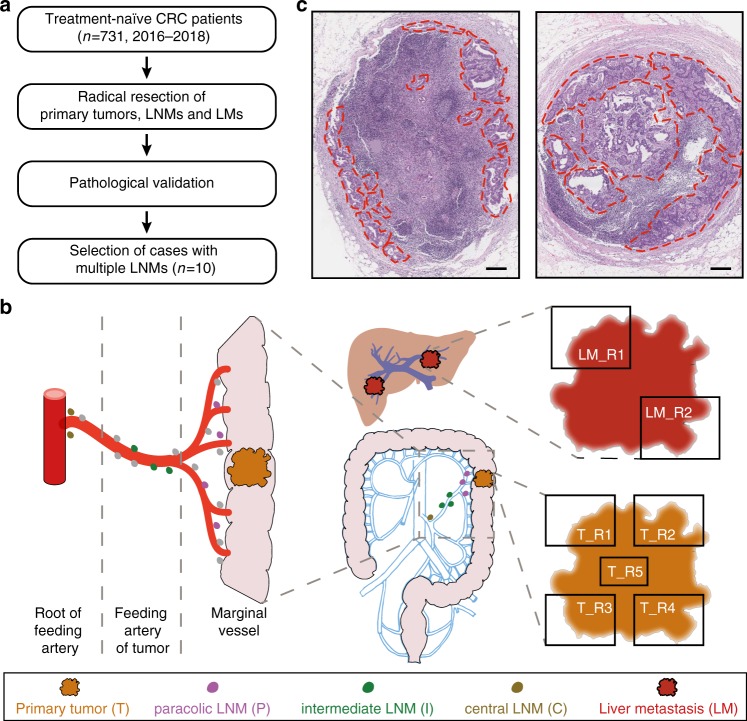
Research strategy. **a** Case screening pipeline. Ten treatment-naive CRC patients with multiple LNMs were selected for this study. **b** Schematic showing CRC patients with LNMs and LMs. Spatial distribution and classification of paracolic (purple), intermediate (green), and central (brown) LNMs are shown on the left. Lymph nodes without tumor cell are colored gray. Multiple tumor regions (2–5 regions according to tumor size) were isolated from the primary tumor and liver metastasis. **c** Two representative whole-slide LNM sections stained with hematoxylin and eosin showing the tumor regions isolated by laser capture microdissection. Tumor regions (marked by red dashed lines) were marked by pathologists. Scale bar = 2.5 mm.

A total of 10 treatment-naive microsatellite-stable patients with CRC were prospectively enrolled in the present study from 2016 to 2018 (Fig. 1a, Supplementary Fig. 1). Detailed clinical information is described in Supplementary Data 1. All the tumor-positive lymph nodes, i.e., lymph node metastases (LNMs), were collected from these patients. To gain a higher resolution of the metastatic network of CRC, LNMs were further classified into paracolic (denoted as P1, P2, etc.), intermediate (denoted as I1, I2, etc.), and central (denoted as C1, C2, etc.) subgroups according to their spatial distance from the primary tumor (Fig. 1b, see Methods)^30^. Given that intratumor heterogeneity is well characterized in CRC, we isolated multi-region samples from the primary tumor (2–5 regions) and liver metastases (2 regions) according to the tumor size in order to capture subclones that may otherwise be missed by single-region sequencing (Fig. 1b). Laser capture microdissection was used to purify tumor cells from the massive immune-cell background in LNMs (Fig. 1c).

Collectively, we retrieved high-quality exome sequencing data at an average depth of 264X from 104 samples (Supplementary Data 2), including 28 primary tumors, 32 paracolic LNMs, 18 intermediate LNMs, 5 central LNMs, 11 LMs, and 10 matched normal tissues (Supplementary Data 1). Among these, 93 samples were isolated from fresh frozen tissues and 11 samples from formalin-fixed paraffin-embedded (FFPE) tissues (Supplementary Data 2). The 55 LNMs (32 paracolic + 18 intermediate + 5 central LNMs) accounted for 72% of the total LNMs from 10 patients (Supplementary Data 1). In Patient_7, Patient_9, Patient_12, Patient_14, and Patient_15, 100% of LNMs were covered.

### Genomic landscape of 10 CRC patients

**Fig. 2:**
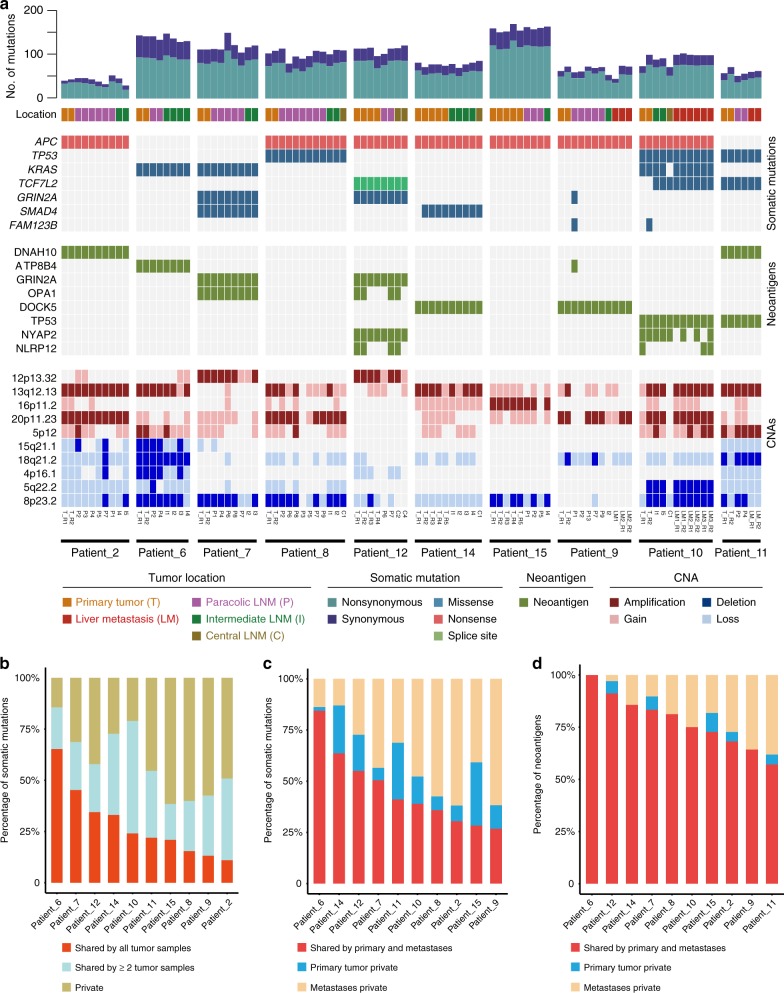
Genomic landscape of 10 CRC patients. **a** The top panel shows the number of mutations identified in each tumor sample. The spatial location of each tumor sample is denoted below. The second panel details seven recurrent driver genes (mutated in ≥2 patients) with the mutation type indicated. The third panel details recurrent genes associated with putative neoantigens. The bottom panel shows recurrent CNAs: dark red for amplifications (CN ≥ 4), light red for gains (2 < CN < 4), dark blue for deletions (CN = 0), and light blue for losses (0 < CN < 2). **b** Percentage of mutations shared by all the tumor samples, two or more tumor samples, and private to only one tumor sample in each patient, respectively. **c** Somatic mutations and **d** Neoantigens shared between the primary tumors and metastases.

A total of 2499 somatic mutations, including 1943 non-silent mutations (1596 point mutations and 347 indels) and 556 silent mutations, were identified across the 94 tumor samples (Fig. 2a, Supplementary Data 3). A range of 117–374 non-silent mutations, with a median of 173.5, were identified across patients. The number of mutations was comparable across patients; however, a variable extent of intratumor heterogeneity was observed across patients, since tumor samples from the same patient shared different percentages of mutations (range: 11–65%, median: 23%, Fig. 2b). To explore the potential driver genes mutated in our CRC cohort, we summarized a list of 52 genes reported in two previous large cohort CRC studies: the Cancer Genome Atlas (TCGA cohort^3^) and Memorial Sloan Kettering Cancer Center (MSK cohort^5^) (Supplementary Data 4, see Methods). Among these, seven genes were mutated in two or more cases in our study (Fig. 2a). Well-known driver genes for CRC, such as *APC* (70%), *TP53* (30%), and *KRAS* (30%), were identified. Mutations in *APC* and *TP53* were shared by all the lesions in affected patients.

To compare the immunogenicity across different tumors from the same patient, we also predicted the putative neoantigens based on somatic mutations^31^ (Fig. 2a, Supplementary Fig. 2). A range of 11–78 neoantigens, with a median of 28, were predicted across patients. The number of neoantigens was comparable among the tumors from each patient; however, a variable extent of intratumor heterogeneity was observed across patients, since tumor samples from the same patient shared different percentages of neoantigens (range: 36–100%, median: 48%, Supplementary Data 5). Among those, eight associated genes were recurrently identified, including the potential driver genes, *TP53* and *GRIN2A*. Neoantigens derived from *DNAH10*, *GRIN2A*, *DOCK5*, and *TP53* mutations were shared by all the lesions in affected patients.

We also investigated the recurrent copy number alterations (CNAs) reported by TCGA in our cohort. A total of 10 recurrent regions were selected, including gains (2 < CN < 4) or amplifications (CN ≥ 4) in 5p12, 12p13.32, 13q12.13, 16p11.2, and 20p11.23, as well as losses (0 < CN < 2) or deletions (CN = 0) in 4p16.1, 5q22.2, 8p23.2, 15q21.1, and 18q21.2 (Fig. 2a). While many of these CNAs were shared by all lesions in the same patient, some lesions showed different copy number statuses. For instance, in Patient_7, P7 and I2 had gains in 12p13.32, while other lesions exhibited amplifications in the same region. The difference in the copy number of this region resulted from different CNA events because they showed non-identical breakpoints.

### Genomic comparison between primary tumors and metastases

The number of mutations was comparable across the primary tumors, LNMs, and LMs (primary vs LNM, *p* = 0.51; primary vs LM, *p* = 0.12; LNM vs LM, *p* = 0.24; Wilcoxon rank-sum test, two-sided). No driver genes were identified only in the metastases, which is consistent with previous observations^5,15^. However, variable proportions of somatic mutations were shared between primary tumors and metastases (range: 26.8–84.4%, median: 40%, Fig. 2c). These results indicate that not all CRC patients showed a high level of genomic concordance between the primary tumor and metastases. Similarly, variable proportions of neoantigens were shared between primary tumors and metastases (range: 57–100%, median: 78%, Fig. 2d). Interestingly, the proportion of neoantigens that were shared by the primary and metastases was much higher than that were private to metastases, suggesting that these mutations may be not actually immunogenic.

### Clonal evolutionary history of Patient_8

**Fig. 3:**
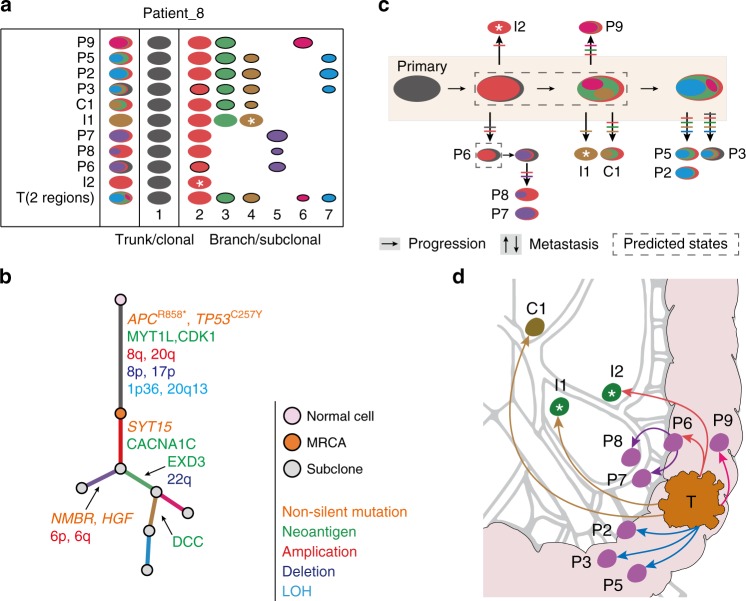
Clonal evolutionary history and parsimonious metastatic map of Patient_8. **a** Oval plots showing the subclonal structure of tumor samples from Patient_8. Each row represents a sample. Ovals in the same color represent the same mutation clusters and are denoted by numbers. The area of each oval is proportional to its CCF value. Subclones are shown with solid borders. Subclonal structures are illustrated by the nested ovals to the left. The mutation cluster clonal in all lesions (cluster 1) is the trunk cluster, representing the most recent ancestor clone (MRCA). Other mutation clusters (clusters 2–7) shared by two or more lesions are defined as branch clusters, representing branch subclones. White asterisks denote monoclonal metastases. **b** Clonal evolutionary tree inferred from the subclonal structure. Lengths of lines are proportional to the number of substitutions in each cluster. Selected aberrations are labeled accordingly. LOH, loss of heterozygosity. **c** Clonal evolutionary history. The dashed box shows the predicted historical states of the primary tumor. Horizontal arrows denote acquisition of new subclones and tumor progression. Vertical fishbone-like arrows denote metastases, which are labeled with involved clone/subclones. Each colored line corresponds to a clone/subclone. **d** Parsimonious metastatic map based on the clonal evolutionary history. Each metastasis is colored by the smallest involved subclone.

To explore the spatiotemporal seeding patterns of LNMs in CRC, we reconstructed the clonal evolutionary history and metastatic routes for each patient. Here, we first demonstrate our analysis strategy with Patient_8, who harbored all three subgroups of LNMs (Fig. 3). Pyclone was adopted to calculate the cancer cell fraction (CCF) of each mutation, which were then grouped into mutation clusters^32^. Since different regions isolated from the primary tumor (here as two regions, T_R1 and T_R2 in Patient_8) collectively reflect its clonal composition, these regions should be taken as a whole rather than separately for subsequent metastasis route analysis. For instance, a mutation cluster that is clonal in T_R1 yet subclonal or absent in T_R2 should be regarded as subclonal for the primary tumor. Thus, a merged mutation CCF value was calculated for the primary lesion as previously described^15^ (Supplementary Data 6, Methods).

A total of seven mutation clusters were identified in Patient_8 (Fig. 3a). The pigeonhole principle was then applied to infer the nesting relationship across the 7 mutation clusters^14,21^, as illustrated by nested ovals (Fig. 3a). Subsequently, a phylogenetic tree was constructed to infer the parent–offspring relationship of mutation clusters (Fig. 3b). Each tumor clone is a group of tumor cells that have identical mutation composition. An offspring clone must inherit all the mutations from its parental clone. To simplify the illustration of tumor clones, we used the color code of the most recent offspring mutation cluster to represent the corresponding tumor clones. For instance, when we refer to the green subclone of Patient_8, we mean tumor cells that have all the mutations in mutation cluster 3 as well as its parental mutation clusters 1 and 2.

The mutation cluster that was clonal in all lesions was defined as the trunk cluster (Fig. 3a, cluster 1, dark gray ovals). The trunk cluster corresponded to the tumor clone that was the parent of all the subclones and was defined as the most recent clone ancestor (MRCA) for Patient_8. Other mutation clusters shared by two or more lesions were defined as branch clusters (clusters 2–7). Notably, cluster 2 (the red ovals) was present but not clonal in all lesions; therefore, it was not a true trunk cluster and should be defined as a branch cluster. These mutation clusters corresponded to different branch subclones. Mutation clusters present in only one lesion were defined as leaf clusters and corresponded to leaf subclones. Leaf subclones were not considered when inferring the clonal evolutionary history and parsimonious metastatic map throughout the text.

The nested ovals of mutation clusters represented the clonal composition of different lesions (Fig. 3a), and the phylogenetic tree of mutation clusters represented the parent–offspring relationship between clones and subclones (Fig. 3b). Acquisition of selected aberrations, including somatic mutations, CNAs, neoantigens, and loss of heterozygosity (LOH), are labeled accordingly on the tree (Fig. 3b). In the MRCA of Patient_8, we identified mutations in *APC* and *TP53*, CNAs in 8p and 8q, and neoantigens derived from *MYT1L* and *CDK1*. In the purple subclone shared by P6, P7, and P8, mutations in *NMBR* and *HGF* and amplification in chromosome 6 were identified.

To infer the clonal evolutionary history and parsimonious spreading routes of LNMs, we assume (1) lymphatic metastases follow a proximal-to-distal pattern due to the unidirectional flow of lymph^33^, and no reverse traffic is considered without definitive evidence; (2) vanished clones due to immune clearance or clonal sweep are not considered^21^; and (3) migrations are rare events during tumor evolution^18^, and each metastasis is generated by only one seeding event. In this scenario, if multiple subclones were shared by two lesions, these subclones migrated jointly in one metastasis event.

Complying with these principles, we predicted the clonal evolutionary history of Patient_8, during which gaining new subclones posited tumor progression and spreading of subclones posited tumor metastases (Fig. 3c). For a certain metastasis, if all the mutation clusters were clonal, the metastasis was seeded by a single clone, defined as monoclonal seeding. For instance, I1 had mutations from clusters 1, 2, 3, and 4, and all mutations were clonal, showing that I1 was a metastasis composed 100% of cells from the brown tumor subclone. Therefore, I1 was generated by the monoclonal seeding of the brown tumor subclone from the primary tumor. Similarly, I2 was seeded by the monoclonal seeding of the red tumor subclone from the primary tumor. In contrast, all other LNMs had one or more subclones. Each metastasis was seeded by multiple subclones, defined as multiclonal seeding. For instance, P9 had 4 mutation clusters, among which clusters 1 and 2 were clonal, while clusters 3 and 6 were subclonal. This suggested that P9 was collectively seeded by the red, green, and magenta subclones from the primary tumor; therefore, P9 was founded by the multiclonal seeding of three different subclones. Similarly, both P5 and P2 resulted from multiclonal seedings of four different subclones (red, green, brown, and blue). Collectively, among the 10 metastatic processes, 2 were monoclonal seedings and 8 were multiclonal seedings.

Finally, we portray the parsimonious metastatic map of Patient_8 (Fig. 3d). Notably, while most metastases were directly seeded by the primary tumor, P7 and P8 were reseeded by P6, indicating that a paracolic LNM can be seeded by another paracolic LNM rather than the primary tumor. Interestingly, two intermediate LNMs and one central LNM were seeded by the primary tumor. A sequential spread from a paracolic LNM to an intermediate LNM or from an intermediate LNM to a central LNM was not observed in this patient.

### Clonal evolutionary history of other patients

**Fig. 4:**
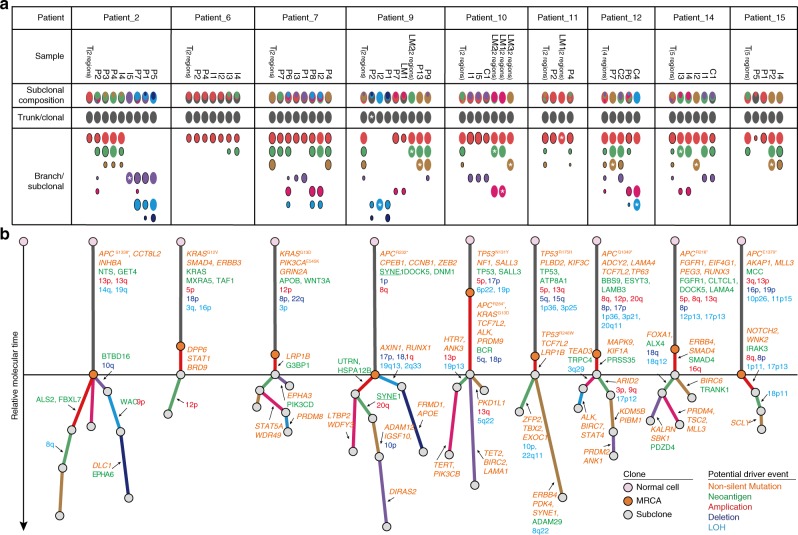
Clonal structures of 9 CRC cases. **a** Oval plots for 9 CRC cases. Clones and subclones are denoted as in Fig. 3. **b** Phylogenetic trees were constructed from the subclonal structure of each patient. The trees were rescaled to fit the plot and are denoted as in Fig. 3b. Detailed genomic events of each patient are shown in Supplementary Figs. 3–11.

Next, we performed the clonal evolutionary analysis for other patients (Fig. 4 and Supplementary Figs. 3–11). Monoclonal seeding was identified in 16 of 61 (26.2%) metastases and in 8 of 10 patients (Figs. 3a and 4a). For instance, in Patient_2, I5 was seeded by a single subclone (purple) from P2. The 16 monoclonal seedings formed 4 paracolic LNMs, 6 intermediate LNMs, 1 central LNM, and 5 LMs. In contrast, 45 of 61 (73.8%) metastases were generated by multiclonal seeding. These results suggest that multiclonal seeding was the major form of metastasis in these 10 CRC patients.

**Fig. 5:**
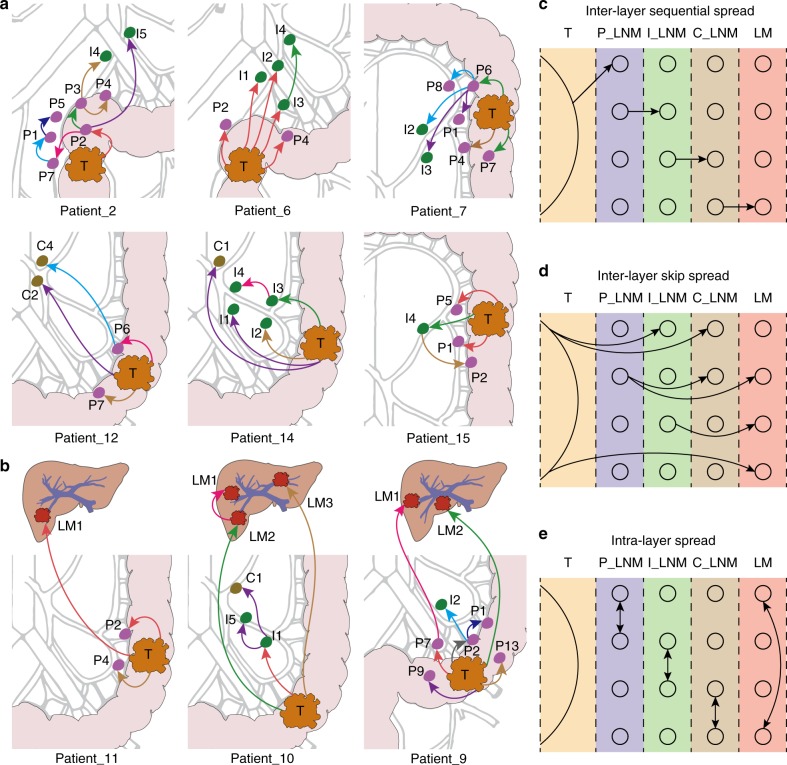
Parsimonious metastatic maps and three modes of metastasis in CRC. **a**, **b** Parsimonious metastatic routes for nine patients. Metastatic routes between lesions are denoted by arrows and colored according to the smallest involved subclone (see Fig. 4). For instance, in Patient_2, P2 is seeded by the red subclone in the primary tumor, thus this route is labeled in red. P3 was then seeded by the red and green subclones from P2, so this route is labeled in green, since the green subclone was smaller. Three metastatic modes were extracted from 61 metastatic events: **c** inter-layer sequential spread, **d** inter-layer skip spread, and **e** intra-layer spread. The metastatic network is dissected into five layers, spanning from T to P, I, C, and LM. The arrows indicate metastatic routes.

Subsequently, we built parsimonious metastatic maps for all patients (Fig. 5a, b). In total, we identified 61 metastatic routes, among which 38 (62.3%) were initiated from the primary tumor, 22 (36.1%) from LNMs, and 1 from a LM (1.6%). These results show that metastasis-to-metastasis spread was prevalent in these CRC patients. Notably, LNMs that reseeded two or more LNMs were identified in five patients. In Patient_8, P6 seeded P7 and P8. In Patient_9, P2 seeded P1 and I2. In Patient_10, I1 seeded I5 and C1. Interestingly, P6 in Patient_7 seeded 4 other metastases, P1, P8, I2, and I3. In Patient_2, P2 seeded P3, P7, and I5, while P3 seeded P4 and I4. These findings suggest that these LNMs may have acquired substantial metastatic potential; however, no recurrent mutations were identified among these LNMs.

### Spreading routes of liver metastases

Next, we focused on patients with liver metastases (LMs) (Fig. 4b, Supplementary Figs. 6–8). Whether LMs are directly seeded by the primary tumor or reseeded by LNMs is intensely debated^11,13,34^. Interestingly, in Patient_9, P7 and LM1 shared a subclone (magenta) that was not present in other lesions; therefore, LM1 was a secondary metastasis reseeded by P7 (Supplementary Fig. 6). The green subclone in the primary tumor was clonal in LM2. Moreover, LM2 shared no subclones with the LNMs of Patient_9. These results suggest that LM2 was directly seeded by a single subclone (green, monoclonal seeding) from the primary tumor. Therefore, both direct seeding from the primary tumor to LMs and secondary seeding from LNMs to LMs coexisted in this patient.

Patient_10 had three LMs. Interestingly, these three LMs were all from monoclonal seeding. LM3 and the primary tumor shared a subclone (brown) that was not present in other lesions; therefore, LM3 was directly seeded by this brown subclone from the primary tumor. Both LM1 and LM2 shared a green subclone with the primary tumor. Meanwhile, LM1 and LM2 shared a magenta subclone that was not present in the primary tumor or other lesions, and this subclone was clonal in both LM1 and LM2. Therefore, one of the two LMs, LM1 or LM2, was seeded by the primary tumor and then reseeded the other. Since LM1 and LM2 had an identical clonal composition, we cannot further distinguish whether LM1 or LM2 was seeded by the primary tumor. Here, we designated that LM2 was seeded by the primary tumor, then LM1 was reseeded by LM2. This result suggested that a liver metastasis could be seeded by another liver metastasis rather than from either the primary tumor or LNMs. Collectively, our results revealed the complex seeding patterns of liver metastasis in CRC.

### Three spread modes of metastasis in CRC

To better describe the metastatic patterns among all lesions, we dissected the metastatic network into five layers: T (primary tumor), P (paracolic LNMs), I (intermediate LNMs), C (central LNMs), and LMs (liver metastases). Any single possible metastatic route belongs to one of the following three modes: inter-layer sequential spread, inter-layer skip spread, and intra-layer spread (Fig. 4c–e). Of the 61 metastatic routes identified among these 10 patients, 27 (44.3%) were inter-layer sequential spread, as exemplified by T to P, P to I, I to C, and C to LM; 21 were inter-layer skip spread (34.4%), as exemplified by T to I, T to C, T to LM, P to C, P to LM, and I to LM; and 13 (21.3%) were intra-layer spread, as exemplified by P to P, I to I, C to C, and LM to LM.

The prevalence of inter-layer skip spread, as seen in 9 of 10 patients, demonstrates that many metastases were seeded by tumor cells from distant anatomic sites rather than their closest neighbors. These findings are in agreement with reports that metastasis does not necessarily follow a stepwise progression model^35–37^, which is consistent with Naxerova et al.’s observation that lymphatic and distant metastases can have distinct clonal origins^13,38^. Moreover, subclonal analysis allows us to further distinguish whether a liver metastasis descends from an LNM or the primary tumor when the liver metastasis and the LNM share a common origin^34^.

## Discussion

Understanding how different lymphatic metastases evolve and spread is critical to tumor staging and therapeutic choice in CRC patients. To comprehensively profile the metastatic routes in CRC, we achieved the highest reported harvest rate of clinically visible LNMs and portray a detailed lymphatic metastatic map of CRC that summarizes three diverse modes of metastases across five layers of the metastatic network. To increase our detection sensitivity of minor subclones in the primary tumor, we have used multi-region sequencing to capture subclones in the primary tumor and LMs that may otherwise be missed by single-region sequencing. Although we cannot completely rule out the possibility that there are some minor subclones in the primary tumor, we believe that our multi-region sampling strategy is a practical way to capture the subclonal composition of the primary tumor. Our results show that while primary-to-metastasis was the major form of metastatic seeding (38/61, 62.3%) in CRC, metastasis-to-metastasis seeding was identified in 9 of 10 patients, accounting for 37.8% (23/61) of all seeding events. In this regard, radical surgical resection of LNMs could benefit most patients in preventing tumor progression.

Detailed clonal comparison between LNMs and LMs was performed in three CRC patients. Interestingly, we identified an LM-to-LM seeding in Patient_10 and coexistence of primary-to-LM and LNM-to-LM in Patient_9. These results suggest that distant metastases may be spread from any tumor site. Whether these distinct spreading patterns result from different genetic alterations remains to be explored. Interestingly, recent studies in mouse models have demonstrated that certain LNMs reseed distant metastases via the blood vessels in lymph nodes rather than via the lymph, which had been generally assumed^39,40^. Our observation of frequent inter-layer skip spread among CRC patients may be explained by this model. Collectively, this evidence reveals the complex seeding patterns of metastases in CRC.

Importantly, our results show that not all LNMs have the same metastatic potential. The existence of certain LNMs that exhibit higher metastatic potential implies that identification and eradication of those LNMs, if possible, may need to be prioritized in future clinical practice. However, we did not identify any genomic alterations or clinical variables that are enriched in these LNMs. Since we only have exome sequencing data, whether these LNMs exhibit distinct noncoding alterations or transcriptomic and proteomic aberrations remains to be explored. A previous longitudinal analysis of metastases in CRC proposed a parallel selection model explaining the role of immunoediting in metastases^41^. In their model, non-recurrent clones are immunoedited, while recurrent clones are immunoprivileged; therefore, LNMs with a higher metastatic potential may acquire immune escape features. However, the metastases harvested at different time points in their study shared very rare mutations (*n* = 0 in P210, and *n* = 6 in P45) as well as neoantigens^41^. This is different from our observation that synchronous metastases shared many mutations and neoantigens with the primary tumor, implying that metastases inherit most neoantigens from the primary tumor and continually acquire new neoantigens during tumor progression. Neoantigen depletion in metastasis was not observed in the present study. One possibility for this is that the metastases that could not escape from the immune surveillance had been eradicated and were therefore not harvested. The manner in which tumor cells interact with immune cells in the metastatic seeding network warrants further study^42^.

Furthermore, the existence of clonal neoantigens in 10 CRC patients indicates that future personalized vaccine therapies should target these clonal neoantigens to elicit a T-cell response that targets all cancer cells, covering both the primary tumor and metastases^43^. As observed in the present study and previous reports^8–10,44^, different regions from the primary tumor and matched primary-metastasis pairs showed a variable extent of intratumor heterogeneity; therefore, sequencing only one region of the primary tumor may bias the determination of clonal neoantigens. Low or no response to personalized cancer vaccines may be explained by selecting and targeting subclonal neoantigens, since only a subset of tumor cells would be targeted by T cells.

Overall, our study only involved 10 patients; therefore, future studies involving a greater number of patients and ideally a 100% harvest rate of clinically visible LNMs are needed to validate our results. Studies integrating multi-omics data are required to characterize the biomarkers of LNMs with a higher metastatic potential. Single-cell sequencing has been used to investigate tumor^45^ and immune^46^ cells in CRC. Future studies analyzing both tumor and immune cells in primary-LNM-LM trios using single-cell sequencing could resolve the intratumor heterogeneity and clonal composition at a higher resolution^47^, providing further clinical insight to prevent tumor cells from spreading to LNMs and LMs in CRC patients.

## Methods

### Spatial classification of LNMs

Lymph node metastases (LNMs) were classified into three subgroups based on the spatial information retrieved from surgical records: LNMs located along the bowel and the mesenteric marginal vessel were determined as paracolic LNMs (denoted as P1, P2, etc.); LNMs located along the feeding artery of the tumor were determined as intermediate LNMs (denoted as I1, I2, etc.); LNMs located at the root of the feeding artery were determined as central LNMs (denoted as C1, C2, etc.).

### Patient selection

The present study was approved by the Institutional Review Board of Cancer Hospital, Chinese Academy of Medical Sciences (CHCAMS, Beijing, China). We performed a prospective screen of colorectal cancer (CRC) patients who underwent radical resection in the Department of Colorectal Surgery, CHCAMS from October 2016 to October 2018. We applied a series of screening steps to select cases for this study: (1) 497 sporadic, microsatellite-stable (MSS) patients with informed consent were selected from 731 treatment-naive patients; (2) 147 patients diagnosed with more than two LNMs were then selected; (3) all LNMs were classified into three subgroups: paracolic, intermediate, and central LNMs (detailed above). A total of 29 patients with LNMs covering 2 or 3 subgroups of LNMs were selected (Patient_11 had only paracolic LNMs, but they were included due to the existence of liver metastasis); (4) 13 patients with samples that had sufficient and good-quality DNA were selected; (5) 3 patients with low purity of the primary tumor were excluded (<20 somatic mutations detected). Finally, 10 CRC patients were selected. Liver metastases (LMs) were available in 3 of the 10 patients. All metastases included in the present study were synchronous metastases.

### Sample collection

For the primary tumors and LMs, multiple tumor regions were isolated to reduce the potential influence of intratumor heterogeneity on clonality inferences. For each primary tumor, 2–5 regions were isolated based on the tumor size. Two regions were isolated from each LM of the three patients with LMs. Adjacent non-tumorous tissue samples were isolated from the 10 subjects as control samples. All tumor samples were isolated from viable regions and confirmed to contain more than 70% tumor cells, and control samples were confirmed to be free of tumor cells.

### Laser capture microdissection

For LNMs, laser capture microdissection (LCM) was performed to isolate tumor cells from the dominant immune background. Tissue sections of LNMs were cut consecutively into 1–2 3-μm diagnosis slides and 5–10 10-μm isolation slides. All slides were sent for H&E staining. High-resolution images were captured from the diagnosis slides. The tumor area was confirmed by an experienced histologist on all slides and isolated from the isolation slides by LCM using a Leica LMD7000 Microsystem. The isolated samples were preserved in 200-μL PCR tubes and sent for subsequent DNA isolation.

### DNA isolation

Primary tumors, liver metastases, and normal controls were stored as fresh frozen tissue, and DNA was extracted using the DNeasy Blood & Tissue Kit (Qiagen). For the lymph node metastases stored as fresh frozen tissue, tumor cells were isolated by laser capture microdissection, and DNA was extracted using the QIAamp DNA Micro Kit (Qiagen). For the lymph node metastases stored as FFPE tissue, tumor cells were isolated by laser capture microdissection, and DNA was extracted using the GeneRead DNA FFPE Kit (Qiagen). The DNA concentration was measured using Qubit 3.0 (Invitrogen). Next, the DNA size was checked using a Fragment Analyzer (Advanced Analytical Technologies). In total, the DNA of 94 tumor samples and matched non-tumorous tissue samples from 10 CRC patients were sent for exome library preparation.

### Exome library preparation and sequencing

A total of 200 ng to 1 μg DNA was taken from each sample and sheared into fragments of ~300 bp using a Covaris S2 ultrasonicator (Covaris). The library was constructed using the NEBNext Ultra DNA Library Prep Kit for Illumina (New England Biolabs) according to the manufacturer’s protocol. Exome regions were captured with SureSelect All Exon V6 (Agilent Technologies) according to the manufacturer’s protocol. The post-hybridization amplification product (2 × 150-bp paired-end reads) was quality-checked and sequenced using Illumina HiSeq Xten instruments (Illumina).

### Point mutations and indels

Paired-end reads were aligned to human genome hg19 (UCSC) using the Burrows–Wheeler Aligner with default parameters^48^. Samtools (v0.1.19) was used to convert SAM files to compressed BAM files and sort the BAM files by chromosomal coordinates^49^. The reads were realigned to the genome using the Genome Analysis Toolkit (GATK 2.1–8) based on dbSNP 135 (www.ncbi.nlm.nih.gov/projects/SNP/)^50^. PCR duplicates were marked with Picard (v1.76), and the sorted, marked BAM files were realigned using the Genome Analysis Toolkit at intervals with indel mismatches. Point mutations were called using Mutect (v1.1.4)^51^ with the following filtering criteria: (1) at least 10X coverage was required in the normal sample of each patient bearing at most 1X mutation coverage; (2) at least 10X total coverage was required in tumor samples in which over 3X mutation coverage was required; (3) reads with Phred quality below 20 at each variant position were excluded. For indels, we took the intersection of variant calls from GATK Unified Genotyper and Varscan2 (v2.3.9).

All variants were annotated using the Ensembl Variant Effect Predictor v96 (https://www.ensembl.org/info/docs/tools/vep/) incorporating COSMIC v64 (http://www.sanger.ac.uk/genetics/CGP/cosmic/), dbSNP build 137 (http://www.ncbi.nlm.nih.gov/sites/SNP), and the National Heart, Lung, and Blood Institute Exome Sequencing Project (https://evs.gs.washington.edu/EVS/) annotations.

### Copy number alterations

Sequenza (v2.1.1) was used to call copy number alterations (CNA) while considering both ploidy and cellularity^52^. Briefly, we used BAM files from the WES data of each tumor and the paired normal samples as input to calculate the depth ratio, which was normalized based on both GC content bias and the data ratio. To acquire segmented copy numbers and estimate cellularity and ploidy, the following parameter settings were used: breaks.method = ’full’, gamma = 40, kmin = 5, gamma.pcf = 200, and kmin.pcf = 200. For each tumor sample, the copy numbers of segments were then divided by ploidy following log2 transformation. After filtering out segments smaller than 500 kb, copy number states were determined for each segment. Copy number gains and losses were defined as at least one copy more and one copy less than the estimated ploidy, respectively. Among these gains and losses, amplifications were defined as four or more copies more than the ploidy, whereas deletions were defined as total deletion of the segment. Sex chromosomes were excluded from this analysis.

CNVkit (v0.9.2) was also performed with default parameters on the paired tumor-normal WES data^53^. After segmentation, the absolute integer copy number of each segment was estimated using the threshold and clonal methods. LOH was called by VarScan2^54^.

### Quality control of FFPE samples

In this project, we collected a total of 33 FFPE samples. For FFPE samples, we first checked the DNA size using Fragment Analyzer, and only those with a peak size >4000 bp were kept. Second, DNA integrity was evaluated by PCR with a set of custom-designed primers randomly selected from the genome, generating products ranging from 100 to 500 bp (Supplementary Data 2). FFPE DNA that could not produce PCR products longer than 200 bp was excluded. As a result, 22 FFPE samples were excluded, and the remaining 11 were sent for subsequent library preparation and sequencing. After calling the point mutations and indels, potential C>T/G>A artefacts with bias in read pair orientation were further filtered as previously described^15,55^.

### Determination of putative neoantigens

HLA typing was performed using POLYSOLVER (v4)^56^. The identification of putative neoantigens was performed with pVAC-seq (v1.5.6) using the NetMHCpan (v4.0) algorithm^31^. Candidate neoantigens with a predicted binding affinity of <100 nM were considered to have high affinity.

### Cancer cell fraction analysis

The cancer cell fraction (CCF) of somatic mutations across all regions in each patient was estimated by PyClone (v0.13.0), a hierarchical Bayesian model incorporating local CNAs and tumor purity^32^. We also included mutations that were not located in exome regions in order to improve the sensitivity of the analysis. Sequencing data from multiple samples of the same primary tumor or liver metastasis were combined and analyzed as a single sample. In each patient, mutations accounting for the same proportions of cancer cells across multiple samples were clustered into clones and subclones. The dominant clusters with a mean CCF > 0.85 were classified as clonal, and other clusters with smaller CCFs were classified as subclonal. When CCF ≤ 0.05, the mutation is statistically absent. Mutation clusters containing <5 substitutions or involved mutations localized to a small number of chromosomes were potential false positive calls and excluded from further analysis.

We divided all mutation clusters into three subgroups: (i) trunk/clonal, representing mutations present in all tumor cells in a patient (these mutations were also the most recent common ancestor for this patient); (ii) branch/subclonal, representing subclonal mutations that were shared by multiple tumor regions; (iii) leaf/subclonal, representing subclonal mutations observed in only one tumor region.

### Merged CCF for multi-region samples

Since multiple regions isolated from the primary tumors (2–5 regions) and liver metastases (two regions) collectively reflect their clonal composition, these regions should be taken as a whole rather than separately for subsequent metastasis route analysis. Thus, we first calculated the CCF value for each tumor sample and then computed a merged CCF value for multi-region samples to generate their overall clonal composition. The merged CCF of each SNV was computed as follows:

$${\mathrm{CCF}} = \left\{ {\begin{array}{*{20}{c}} \!\!{\frac{{\mathop {\sum}\nolimits_{i = 1}^k {{\mathrm{CCF}}_i \times d_i} }}{{\mathop {\sum}\nolimits_{i = 1}^k {d_i} }},\,{\mathrm{CCF}} < 1} \\ {1, \quad\quad\quad\quad \,\,\,\,\,{\mathrm{CCF}} \ge 1} \end{array}} \right.$$

For each multi-region sample *i*, *d*_*i*_ is the sequencing depth and CCF_*i*_ is the CCF estimation for this sample. All the merged CCF values are listed in Supplementary Data 6.

### Potential driver genes in CRC

To generate a list of potential driver genes for CRC, we summarized the union of significantly mutated genes reported in two previous large cohort CRC studies: the TCGA cohort (17 genes)^3^ and the MSK cohort (47 genes)^5^ (Supplementary Data 4). *TTN* was excluded as it is one of the longest human genes and mutations in this gene are likely passenger events. Then, 52 genes were selected as potential driver genes for CRC (Supplementary Data 4).

### Genomic landscape

To draw the genomic landscape of 10 CRC patients, we selected 3 categories of aberrations: non-silent mutations, CNAs, and putative neoantigens. For non-silent mutations, we referred to population-level driver mutations. Thus, seven potential driver genes mutated in two or more patients were selected. For CNAs, we selected 10 TCGA reported regions that were recurrent in our cohort. In addition, we also selected eight genes recurrently affected by putative neoantigens.

### Labeling the clonal phylogenetic trees

To label the clonal phylogenetic trees for each patient, we selected four categories of aberrations: non-silent mutations, CNAs, LOHs, and putative neoantigens. For non-silent mutations, we expanded the driver gene list to pan-cancer driver genes, so we selected genes from (1) the above-mentioned 52 potential driver genes; (2) the MSK-IMPACT list of 468 genes (Supplementary Data 4); (3) genes documented in the COSMIC database and related to other types of cancer; (4) genes documented in the Kyoto Encyclopedia of Genes and Genomes (KEGG) cancer pathways. For CNAs, LOHs, and putative neoantigens, we selected some high-confident calls to label the trees.

### Reconstruction of subclonal structure

To reconstruct the subclonal structure of each sample, the relationships among all clones and subclones from all tumor regions were determined jointly based on the pigeonhole principle^14,21^. For any two subclones, the one with a smaller CCF value can either be a descendant (linear relationship) or a brother/sister (branching relationship) to the other. To determine linear and branching relationships, we used the following rules: (i) two subclones were linear if one contained mutations with larger CCFs than the other in all samples, or if the sum of the CCFs in the two subclones was greater than one; (ii) two subclones were branching if the relative CCFs in the two subclones were reversed in some samples; (iii) two branching subclones could not be both linear with an ancestral subclone if the sum of the CCFs of the branching subclones was greater than the CCF of the ancestral subclone. The subclonal structure of each sample is shown by nested ovals. Clonal evolutionary trees for each patient were constructed and labeled with selected aberrations.

### Statistical analysis

Statistical analysis was performed using the R software (v3.5.3). Differences were considered significant at *p* < 0.05.

### Reporting summary

Further information on research design is available in the Nature Research Reporting Summary linked to this article.

## Supplementary information

Supplementary Information

Description of Additional Supplementary Files

Supplementary Data 1

Supplementary Data 2

Supplementary Data 3

Supplementary Data 4

Supplementary Data 5

Supplementary Data 6

Reporting Summary

## Data Availability

The raw sequence data reported in this paper have been deposited in the Genome Sequence Archive in BIG Data Center, Beijing Institute of Genomics (BIG), Chinese Academy of Sciences, under accession numbers HRA000130 [https://bigd.big.ac.cn/gsa-human/s/A7hb3IW7] that are publicly accessible at https://bigd.big.ac.cn/gsa. To access this data, please contact the corresponding authors (Fan Bai, fbai@pku.edu.cn and Haizeng Zhang, haizengzhang@cicams.ac.cn).

## Code availability

Scripts of main analysis steps used in this study are provided at https://github.com/ChongJenniferZhang/CRC_metastases.
