# Supplementary material for: Mapping the spreading routes of lymphatic metastases in human colorectal cancer
Source: Nat Commun. 2020 Apr 24;11:1993. doi: 10.1038/s41467-020-15886-6 (PMC7181746; doi:10.1038/s41467-020-15886-6)
Supplement: Supplementary file 1 — Supplementary Information [file 41467_2020_15886_MOESM1_ESM.pdf]

Supplementary Information

Mapping the spreading routes of lymphatic metastases in human colorectal cancer

Zhang et al.

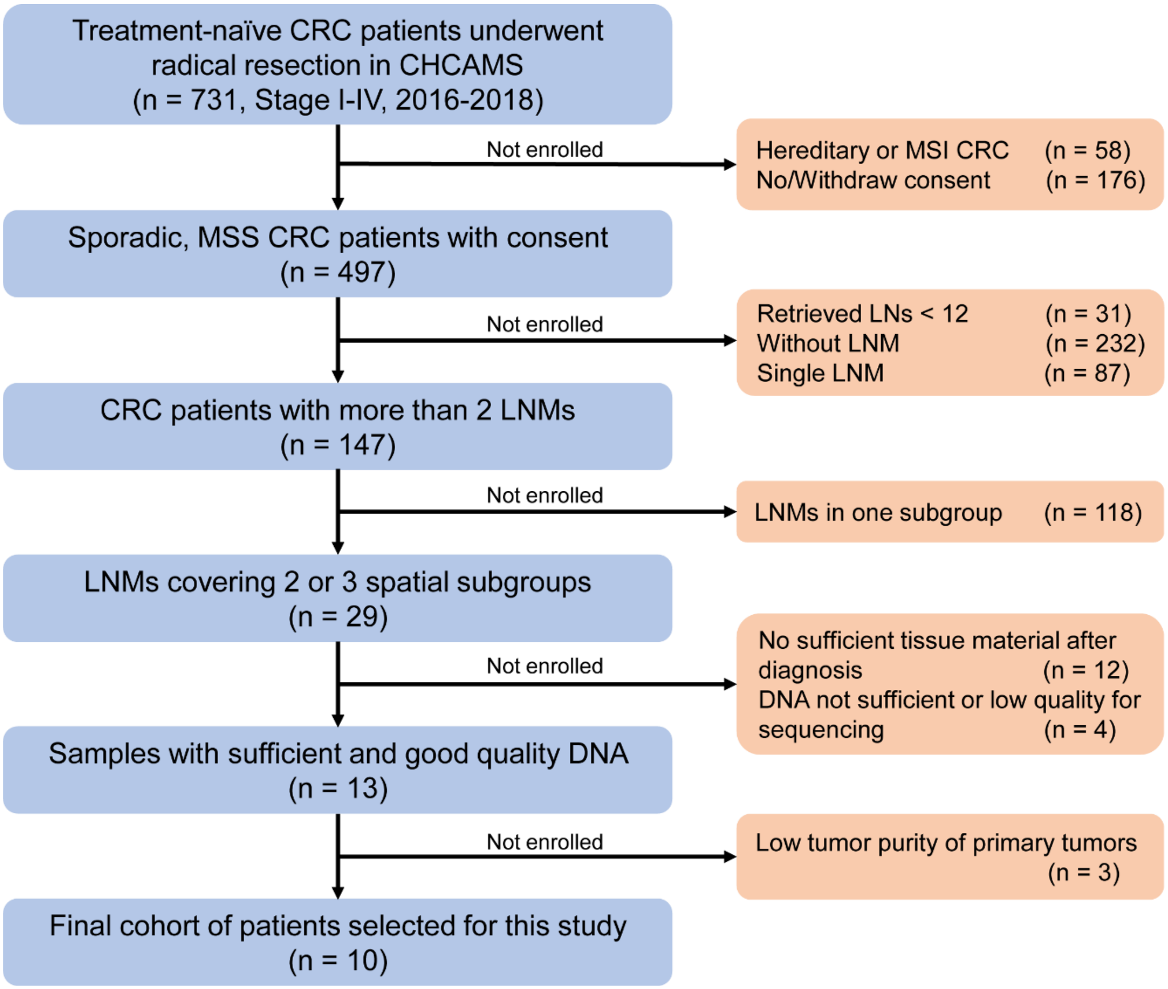

Supplementary Fig. 1: Case screening pipeline of the 10 CRC patients.

CRC, colorectal cancer; MSS, microsatellite-stable; MSI, microsatellite-instable; LN, lymph node; LNM, lymph node metastasis.

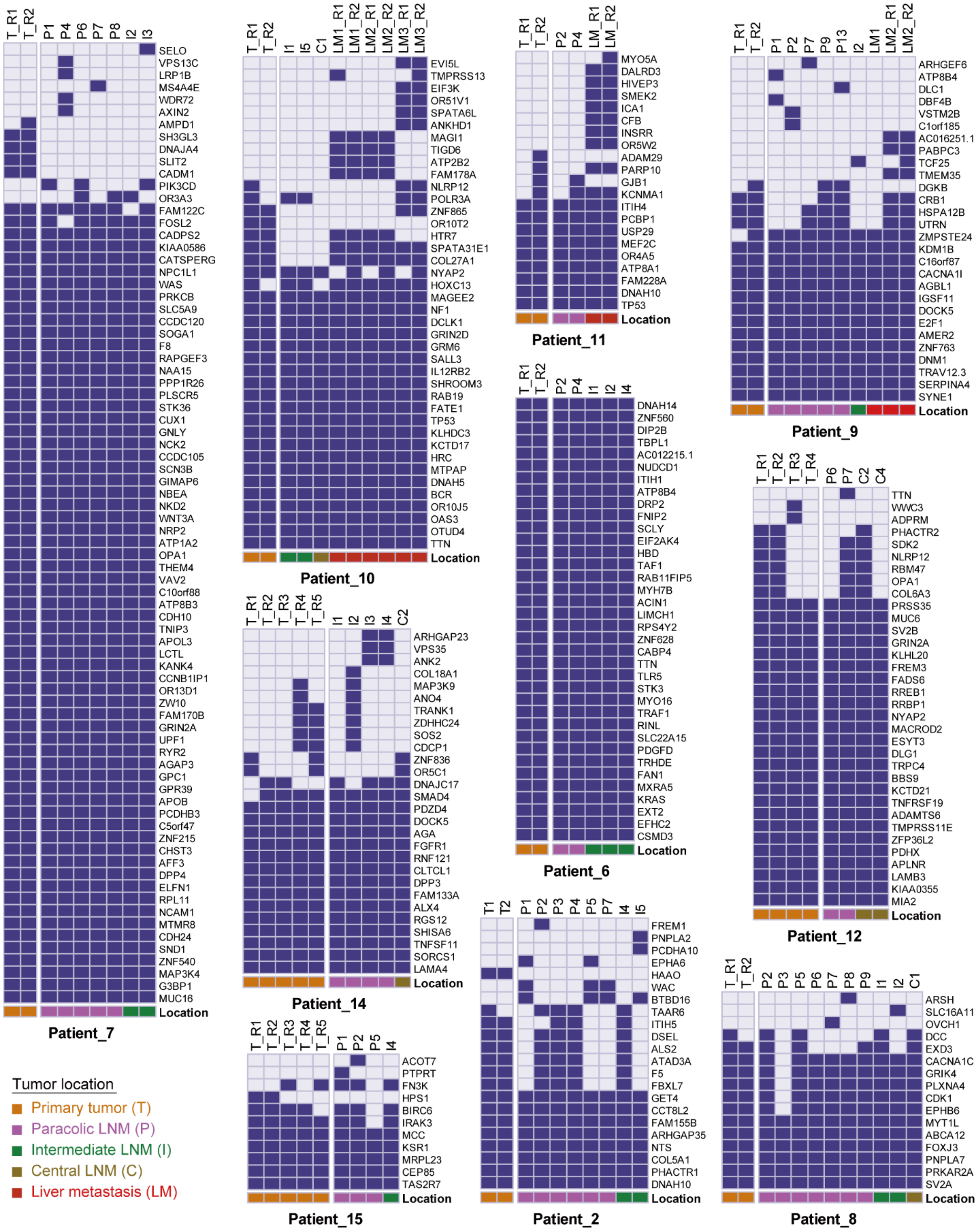
T_R1 T_R2 P1 P4 P6 P7 P8 I2 I3
SELO
VPS13C
LRP1B
MS4A4E
WDR72
AXIN2
AMPD1
SH3GL3
DNAJA4
SLIT2
CADM1
PIK3CD
OR3A3
FAM122C
FOSL2
CADPS2
KIAA0586
CATSPERG
NPC1L1
WAS
PRKCB
SLC5A9
CCDC120
SOGA1
F8
RAPGEF3
NAA15
PPP1R26
PLSCR5
STK36
CUX1
GNLY
NCK2
CCDC105
SCN3B
GIMAP6
NBEA
NKD2
WNT3A
NRP2
ATP1A2
OPA1
THEM4
VAV2
C10orf88
ATP8B3
CDH10
TNIP3
APOL3
LCTL
KANK4
CCNB1IP1
OR13D1
ZW10
FAM170B
GRIN2A
UPF1
RYR2
AGAP3
GPC1
GPR39
APOB
PCDHB3
C5orf47
ZNF215
CHST3
AFF3
DPP4
ELFN1
RPL11
NCAM1
MTMR8
CDH24
SND1
ZNF540
MAP3K4
G3BP1
MUC16
Location
Patient_7
T_R1 T_R2 I1 I5 C1 LM1_R1 LM1_R2 LM2_R1 LM2_R2 LM3_R1 LM3_R2
EVI5L
TMPRSS13
EIF3K
OR51V1
SPATA6L
ANKHD1
MAGI1
TIGD6
ATP2B2
FAM178A
NLRP12
POLR3A
ZNF865
OR10T2
HTR7
SPATA31E1
COL27A1
NYAP2
HOXC13
MAGEE2
NF1
DCLK1
GRIN2D
GRM6
SALL3
IL12RB2
SHROOM3
RAB19
FATE1
TP53
KLHDC3
KCTD17
HRC
MTPAP
DNAH5
BCR
OR10J5
OAS3
OTUD4
TTN
Location
Patient_10
T_R1 T_R2 P2 P4 LM_R1 LM_R2
MYO5A
DALRD3
HIVEP3
SMEK2
ICA1
CFB
INSRR
OR5W2
ADAM29
PARP10
GJB1
KCNMA1
ITIH4
PCBP1
USP29
MEF2C
OR4A5
ATP8A1
FAM228A
DNAH10
TP53
Location
Patient_11
T_R1 T_R2 P1 P2 P7 P9 P13 I2 LM1 LM2_R1 LM2_R2
ARHGEF6
ATP8B4
DLC1
DBF4B
VSTM2B
C1orf185
AC016251.1
PABPC3
TCF25
TMEM35
DGKB
CRB1
HSPA12B
UTRN
ZMPSTE24
KDM1B
C16orf87
CACNA1I
AGBL1
IGSF11
DOCK5
E2F1
AMER2
ZNF763
DNM1
TRAV12.3
SERPINA4
SYNE1
Location
Patient_9
T_R1 T_R2 P2 P4 I1 I2 I4
DNAH14
ZNF560
DIP2B
TBPL1
AC012215.1
NUDCD1
ITIH1
ATP8B4
DRP2
FNIP2
SCLY
EIF2AK4
HBD
TAF1
RAB11FIP5
MYH7B
ACIN1
LIMCH1
RPS4Y2
ZNF628
CABP4
TTN
TLR5
STK3
MYO16
TRAF1
RINL
SLC22A15
PDGFD
TRHDE
FAN1
MXRA5
KRAS
EXT2
EFHC2
CSMD3
Location
Patient_6
T_R1 T_R2 T_R3 T_R4 P6 P7 C2 C4
TTN
WWC3
ADPRM
PHACTR2
SDK2
NLRP12
RBM47
OPA1
COL6A3
PRSS35
MUC6
SV2B
GRIN2A
KLHL20
FREM3
FADS6
RREB1
RRBP1
NYAP2
MACROD2
ESYT3
DLG1
TRPC4
BBS9
KCTD21
TNFRSF19
ADAMTS6
TMPRSS11E
ZFP36L2
PDHX
APLNR
LAMB3
KIAA0355
MIA2
Location
Patient_12
T_R1 T_R2 T_R3 T_R4 T_R5 I1 I2 I3 I4 C2
ARHGAP23
VPS35
ANK2
COL18A1
MAP3K9
ANO4
TRANK1
ZDHHC24
SOS2
CDCP1
ZNF836
OR5C1
DNAJC17
SMAD4
PDZD4
DOCK5
AGA
FGFR1
RNF121
CLTCL1
DPP3
FAM133A
ALX4
RGS12
SHISA6
TNFSF11
SORCS1
LAMA4
Location
Patient_14
T_R1 T_R2 T_R3 T_R4 T_R5 P1 P2 P5 I4
ACOT7
PTPRT
FN3K
HPS1
BIRC6
IRAK3
MCC
KSR1
MRPL23
CEP85
TAS2R7
Location
Patient_15
T1 T2 P1 P2 P3 P4 P5 P7 I4 I5
FREM1
PNPLA2
PCDHA10
EPHA6
HAAO
WAC
BTBD16
TAAR6
ITIH5
DSEL
ALS2
ATAD3A
F5
FBXL7
GET4
CCT8L2
FAM155B
ARHGAP35
NTS
COL5A1
PHACTR1
DNAH10
Location
Patient_2
T_R1 T_R2 P2 P3 P5 P6 P7 P8 P9 I1 I2 C1
ARSH
SLC16A11
OVCH1
DCC
EXD3
CACNA1C
GRIK4
PLXNA4
CDK1
EPHB6
MYT1L
ABCA12
FOXJ3
PNPLA7
PRKAR2A
SV2A
Location
Patient_8
Tumor location
Primary tumor (T)
Paracolic LNM (P)
Intermediate LNM (I)
Central LNM (C)
Liver metastasis (LM)

Supplementary Fig. 2: Putative neoantigens predicted in the 10 CRC patients.

Heatmaps showing the regional distribution of all putative neoantigens; Gene names are listed on the right of each heatmap. Presence (dark purple) or absence (light purple) of each neoantigen is shown. Sample names and locations are indicated at the top and bottom, respectively.

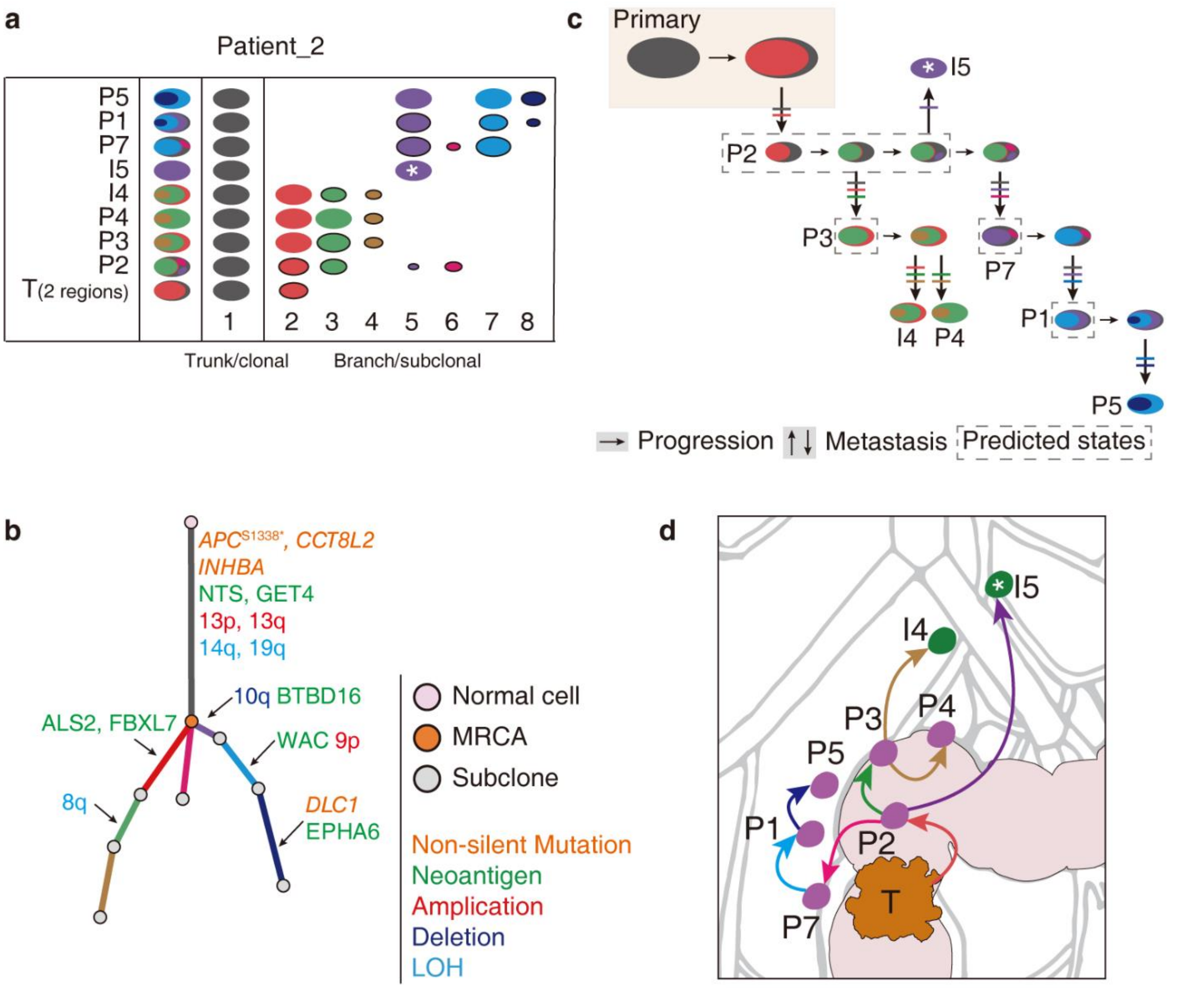

Supplementary Fig. 3: Clonal evolutionary history and parsimonious metastatic map of Patient_2.

a Oval plots showing the subclonal structure of tumor samples from Patient_8. Each row represents a sample. Ovals in the same color represent the same mutation clusters and are denoted by numbers. The area of each oval is proportional to its CCF value. Subclones are shown with solid borders. Subclonal structures are illustrated by the nested ovals to the left. The mutation cluster clonal in all lesions (cluster 1) is the trunk cluster, representing the most recent ancestor clone (MRCA). Other mutation clusters (clusters 2−8) shared by two or more lesions are defined as branch clusters, representing branch subclones. White asterisks denote monoclonal metastasis. **b** Clonal evolutionary tree inferred from the subclonal structure. Lengths of the lines are proportional to the number of substitutions in each cluster. Selected aberrations are labeled accordingly. LOH, loss of heterozygosity. **c** Clonal evolutionary history. The dashed box shows the predicted historical states of the primary tumor. Horizontal arrows denote the acquisition of new subclones and tumor progression. Vertical fishbone-like arrows denote metastases, which are labeled with the involved clone/subclones. Each colored line corresponds to a clone/subclone. **d** Parsimonious metastatic

map based on the clonal evolutionary history. Each metastasis is colored according to the smallest involved subclone.

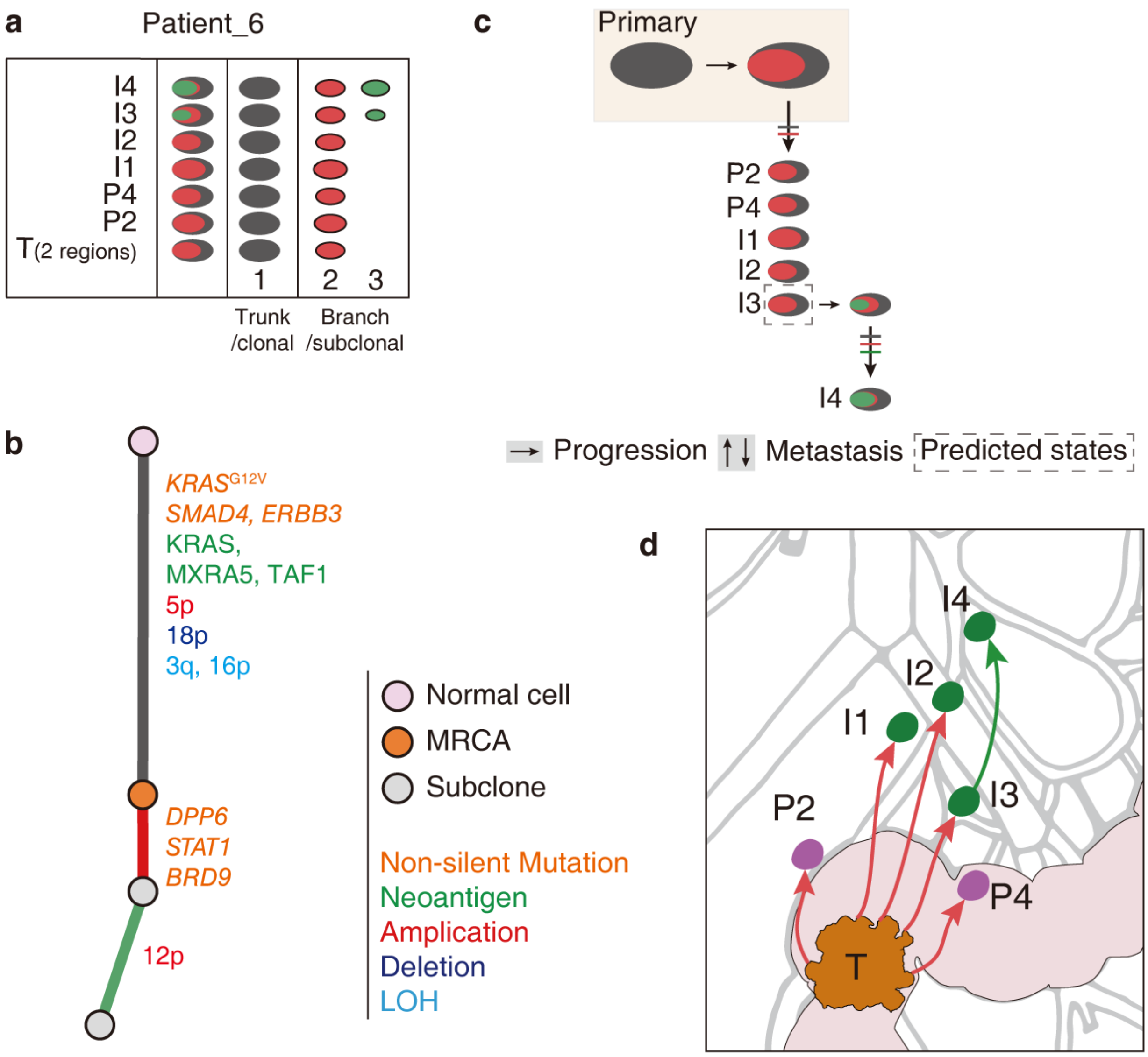

Supplementary Fig. 4: Clonal evolutionary history and parsimonious metastatic map of Patient_6.

a Oval plots showing the subclonal structure of tumor samples. **b** Clonal evolutionary tree inferred from the subclonal structure. **c** Clonal evolutionary history. **d** Parsimonious metastatic map based on the clonal evolutionary history. All panels in this figure are denoted as in **Supplementary Fig. 3**.

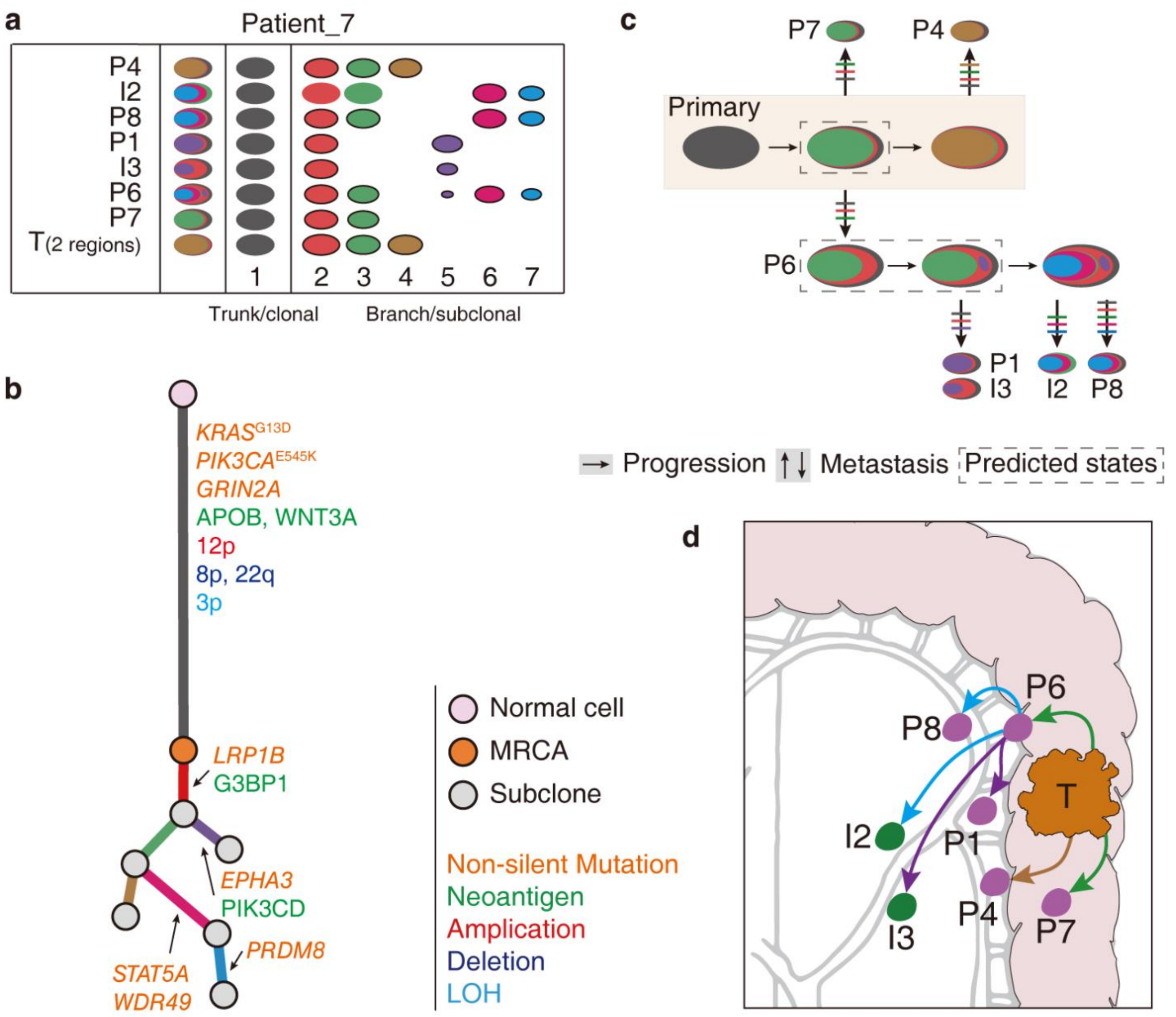

Supplementary Fig. 5: Clonal evolutionary history and parsimonious metastatic map of Patient_7.

a Oval plots showing the subclonal structure of tumor samples. **b** Clonal evolutionary tree inferred from the subclonal structure. **c** Clonal evolutionary history. **d** Parsimonious metastatic map based on the clonal evolutionary history. All panels in this figure are denoted as in **Supplementary Fig. 3**.

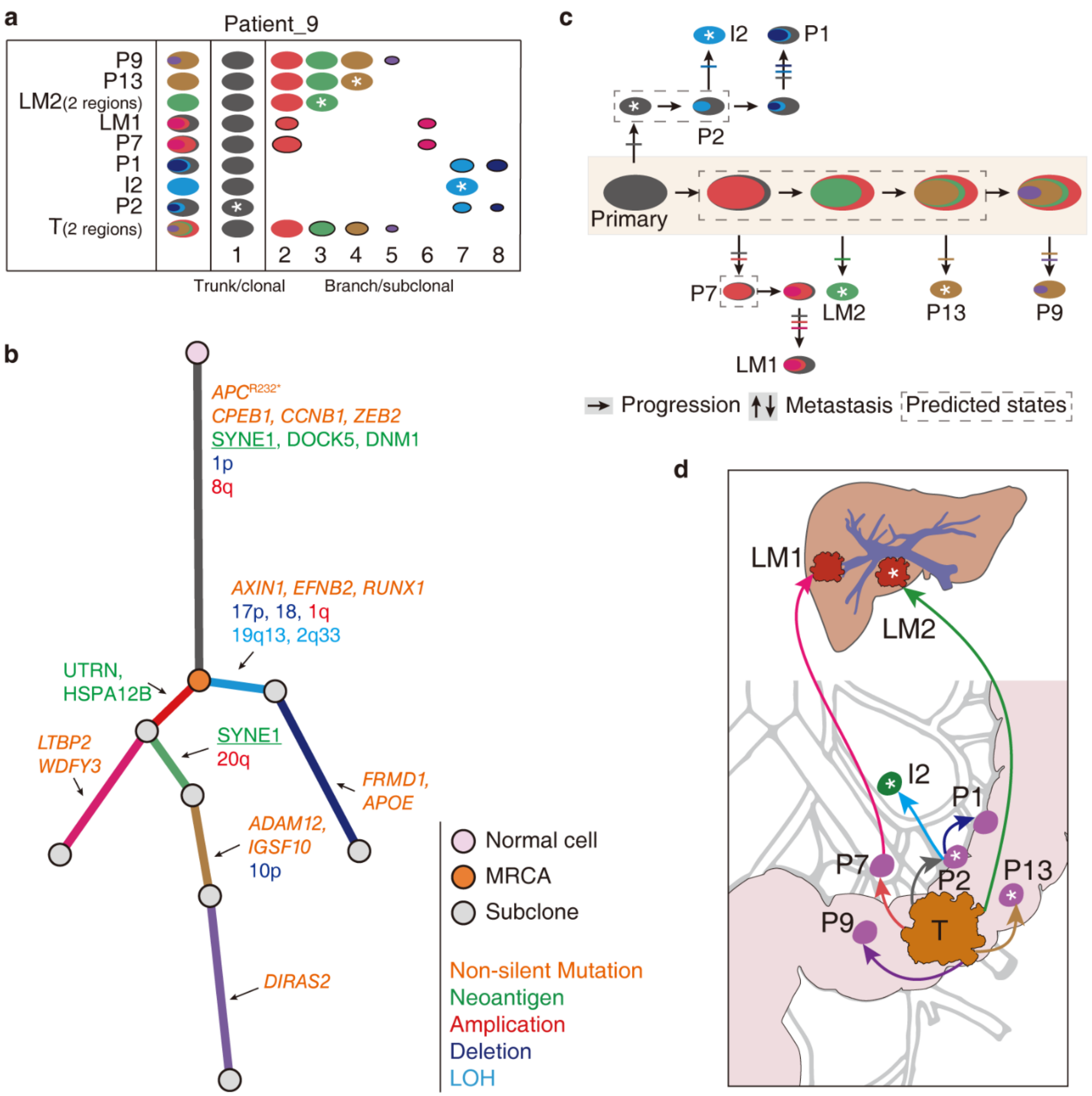

Supplementary Fig. 6: Clonal evolutionary history and parsimonious metastatic map of Patient_9.

a Oval plots showing the subclonal structure of tumor samples. **b** Clonal evolutionary tree inferred from the subclonal structure. Gene marked with underscore denotes convergent evolution. **c** Clonal evolutionary history. **d** Parsimonious metastatic map based on the clonal evolutionary history. All panels in this figure are denoted as in **Supplementary Fig. 3**.

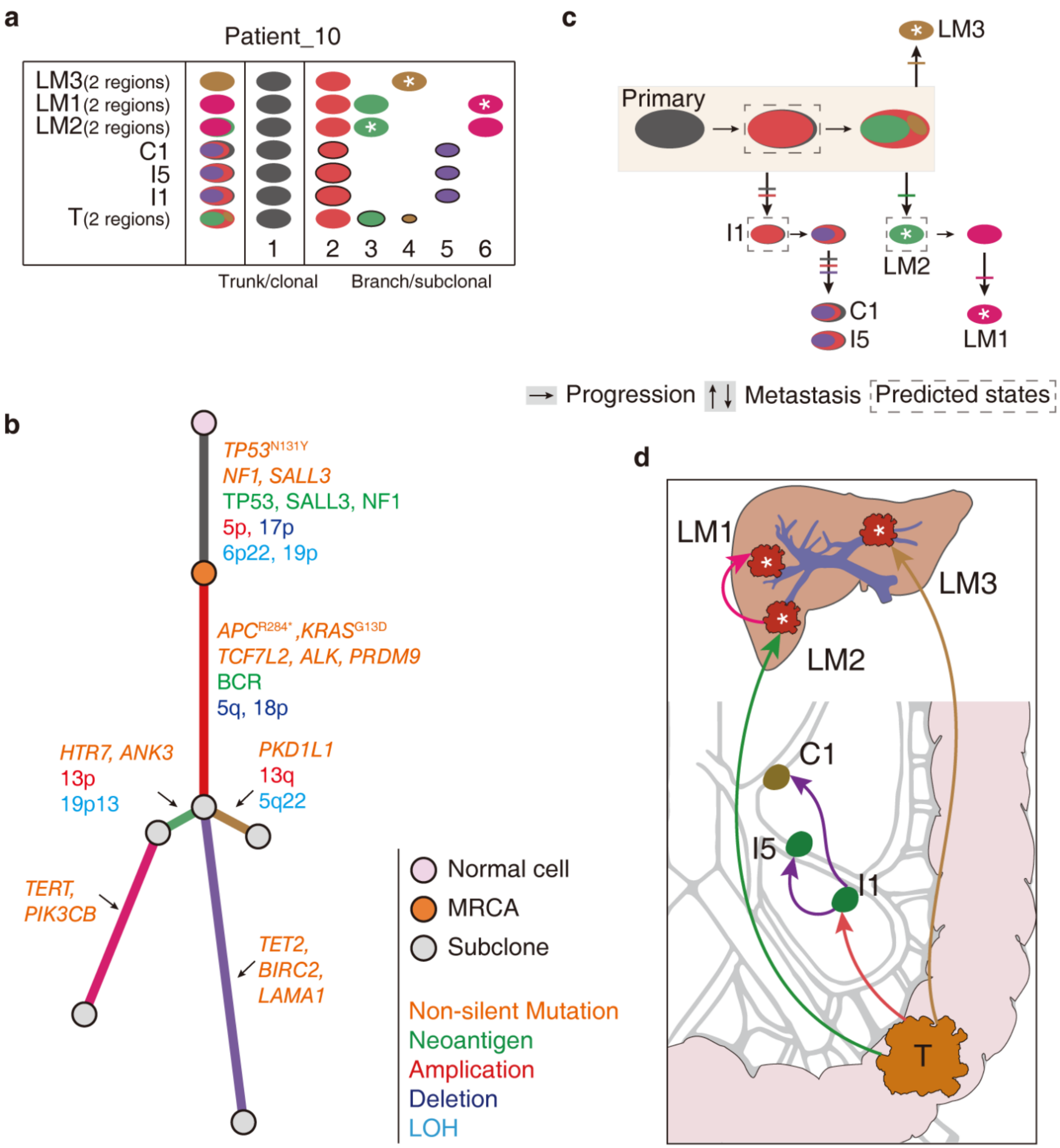

Supplementary Fig. 7: Clonal evolutionary history and parsimonious metastatic map of Patient_10.

a Oval plots showing the subclonal structure of tumor samples. **b** Clonal evolutionary tree inferred from the subclonal structure. **c** Clonal evolutionary history. **d** Parsimonious metastatic map based on the clonal evolutionary history. All panels in this figure are denoted as in **Supplementary Fig. 3**.

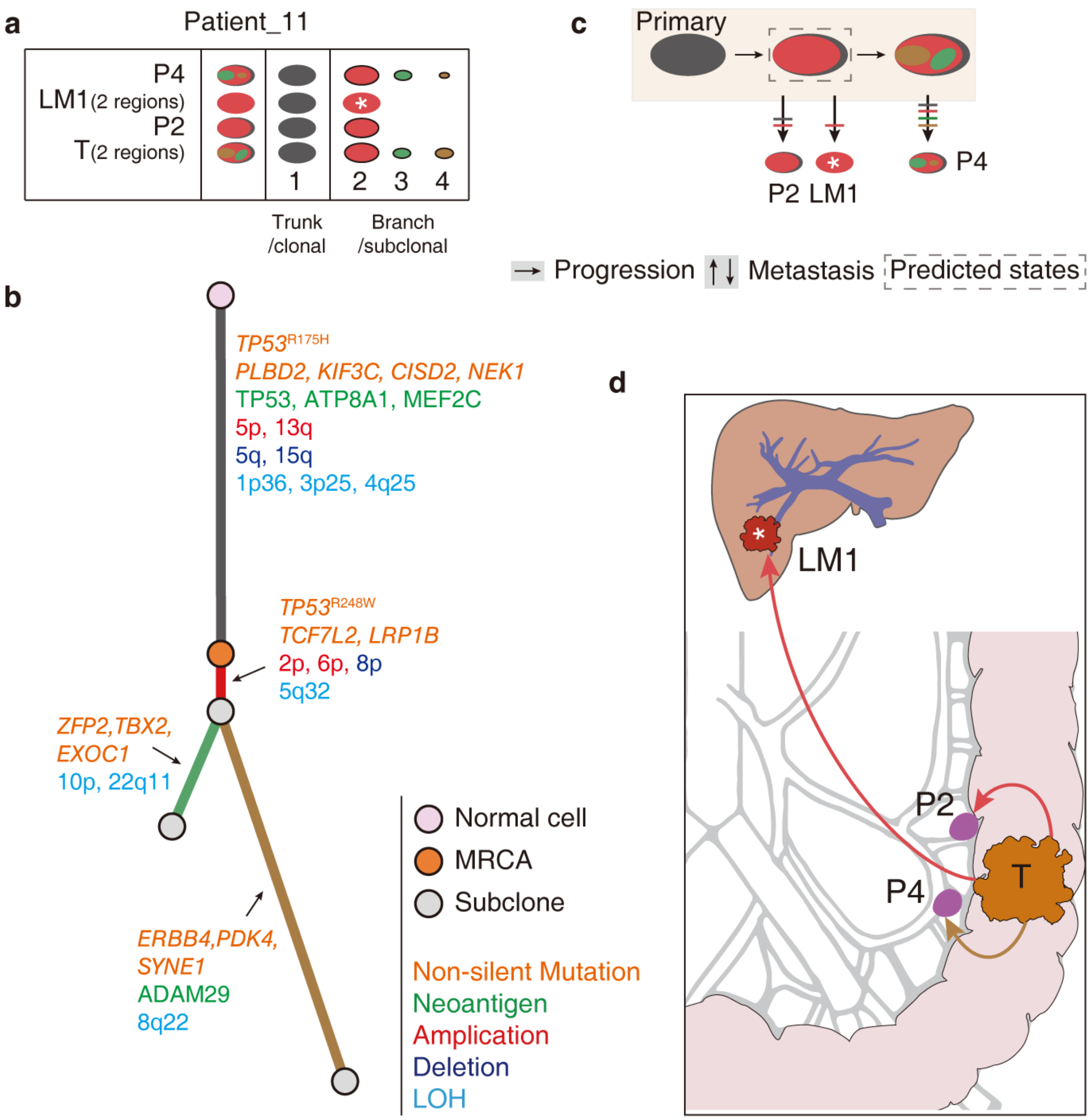

Supplementary Fig. 8: Clonal evolutionary history and parsimonious metastatic map of Patient_11.

a Oval plots showing the subclonal structure of tumor samples. **b** Clonal evolutionary tree inferred from the subclonal structure. **c** Clonal evolutionary history. **d** Parsimonious metastatic map based on the clonal evolutionary history. All panels in this figure are denoted as in **Supplementary Fig. 3**.

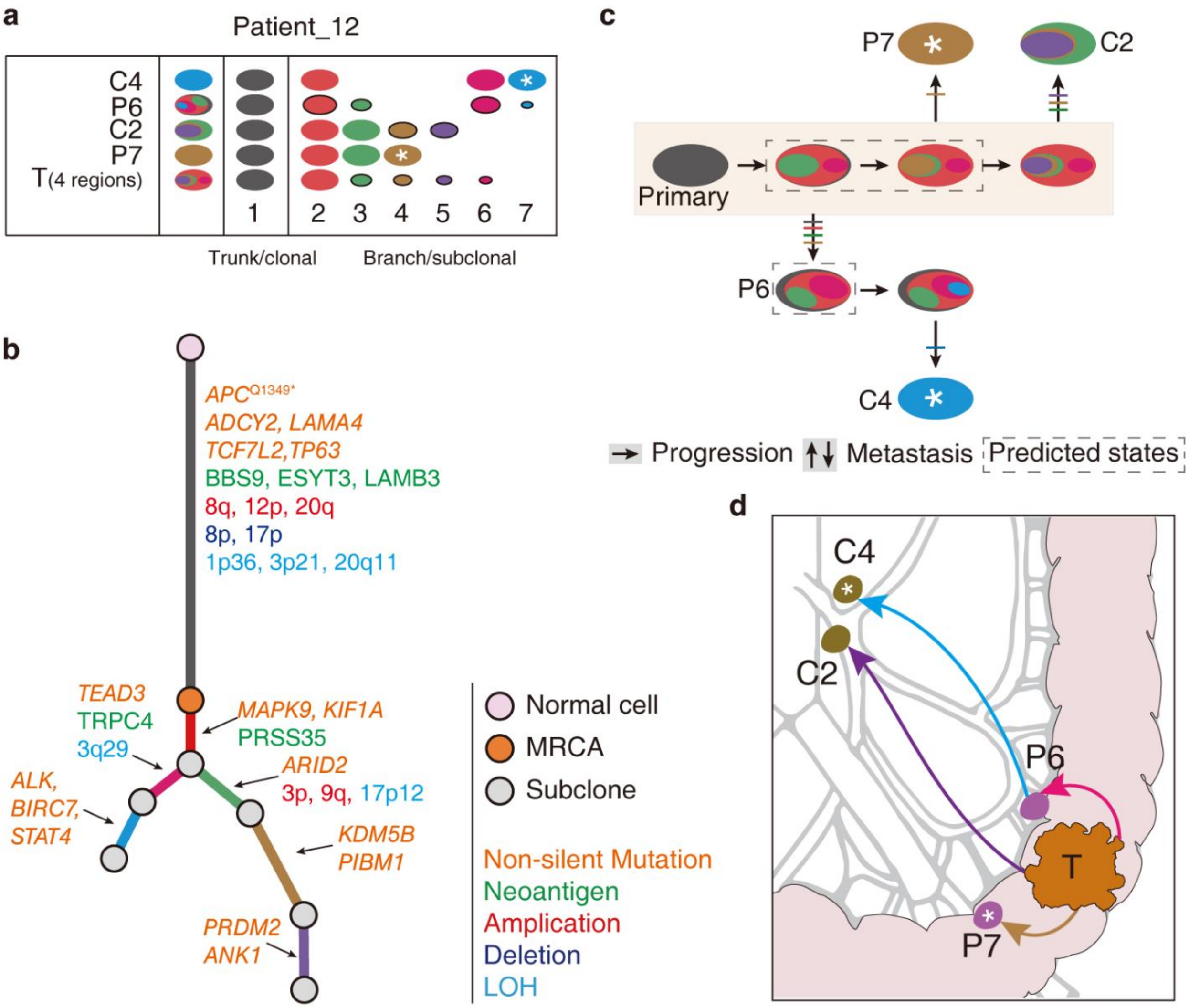

Supplementary Fig. 9: Clonal evolutionary history and parsimonious metastatic map of Patient_12.

a Oval plots showing the subclonal structure of tumor samples. **b** Clonal evolutionary tree inferred from the subclonal structure. **c** Clonal evolutionary history. **d** Parsimonious metastatic map based on the clonal evolutionary history. All panels in this figure are denoted as in **Supplementary Fig. 3**.

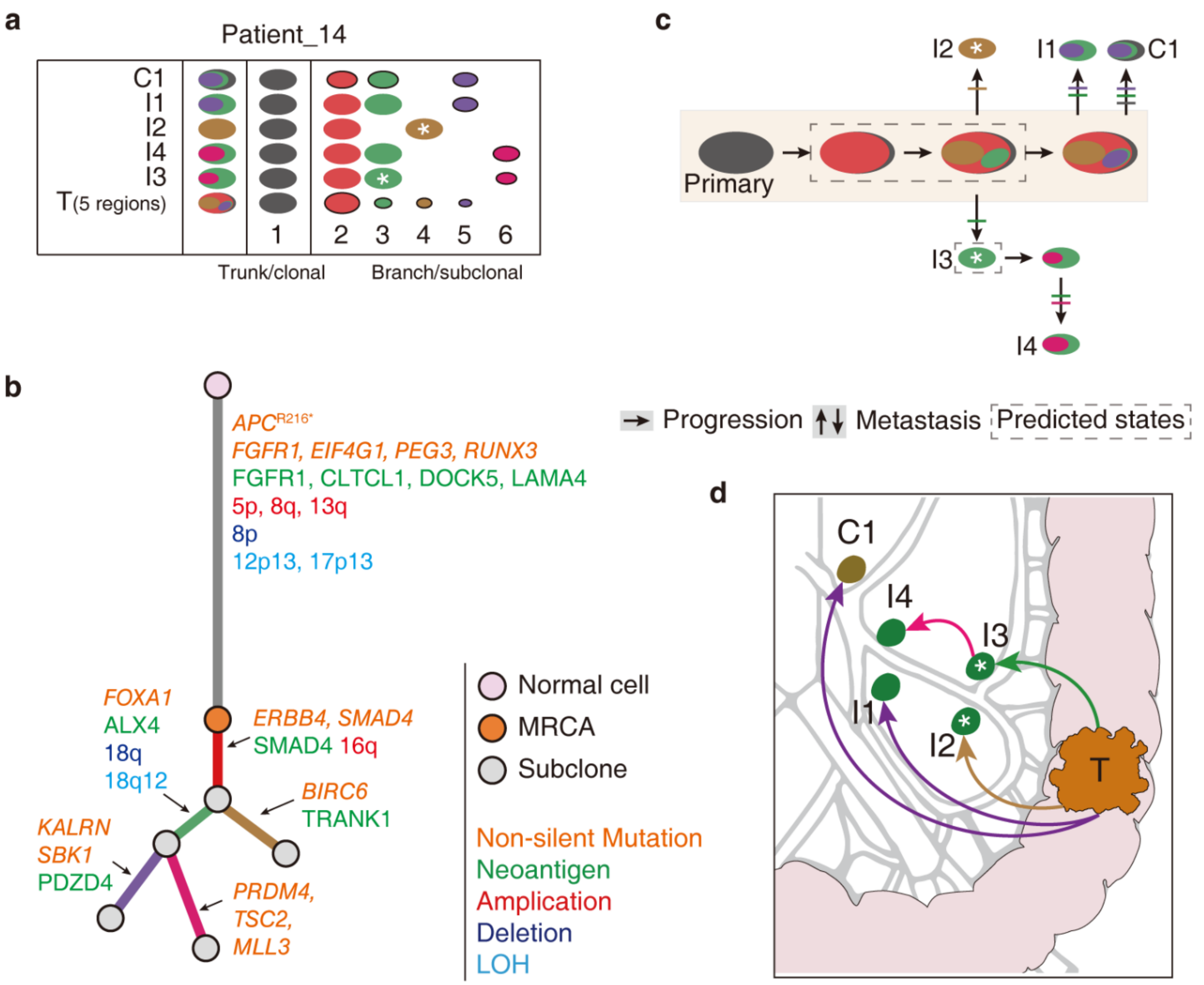

Supplementary Fig. 10: Clonal evolutionary history and parsimonious metastatic map of Patient_14.

a Oval plots showing the subclonal structure of tumor samples. **b** Clonal evolutionary tree inferred from the subclonal structure. **c** Clonal evolutionary history. **d** Parsimonious metastatic map based on the clonal evolutionary history. All panels in this figure are denoted as in **Supplementary Fig. 3**.

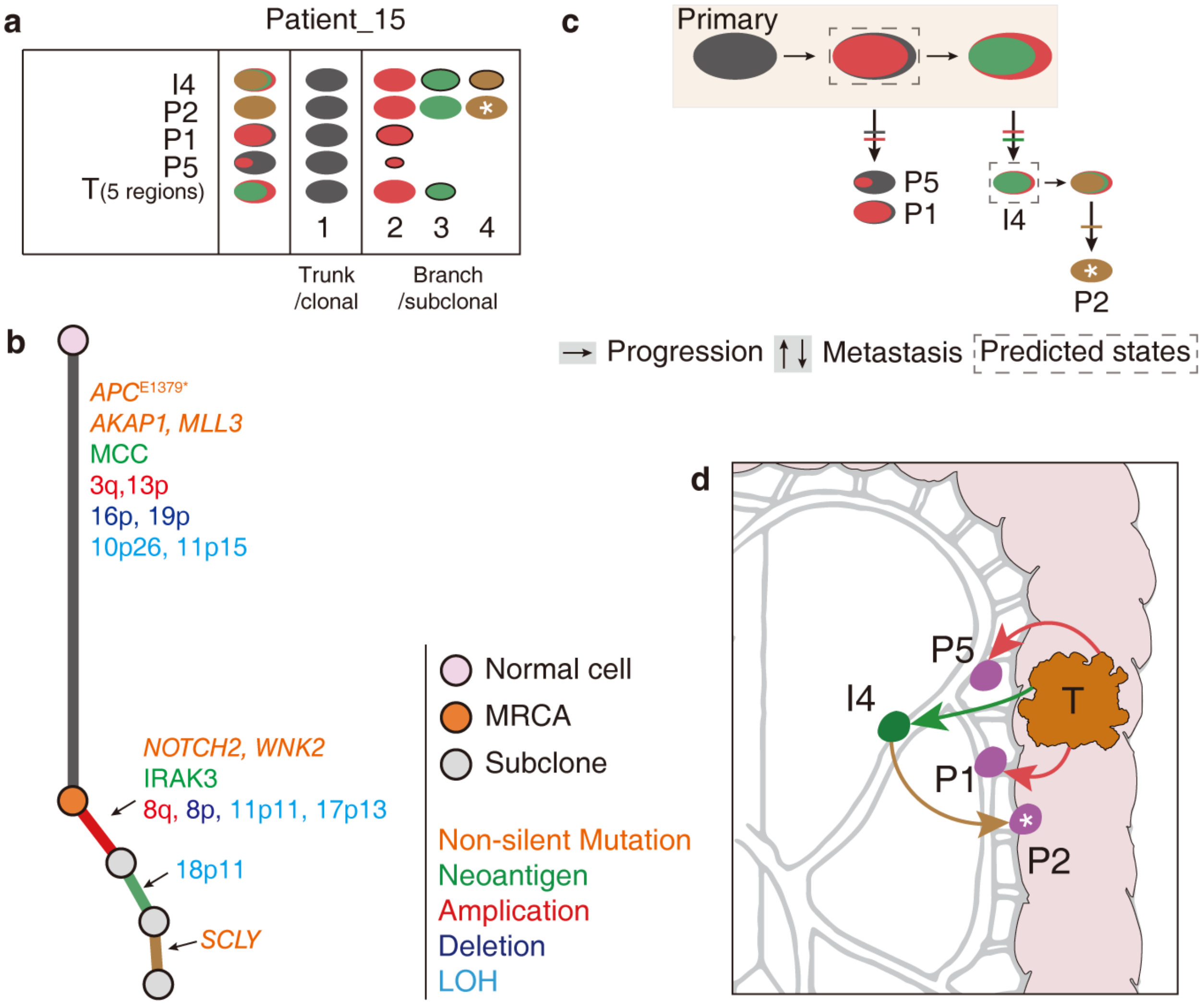

Supplementary Fig. 11: Clonal evolutionary history and parsimonious metastatic map of Patient_15.

a Oval plots showing the subclonal structure of tumor samples. **b** Clonal evolutionary tree inferred from the subclonal structure. **c** Clonal evolutionary history. **d** Parsimonious metastatic map based on the clonal evolutionary history. All panels in this figure are denoted as in **Supplementary Fig. 3**.
