# Supplementary material for: Mapping the spreading routes of lymphatic metastases in human colorectal cancer
Source: Nat Commun. 2020 Apr 24;11:1993. doi: 10.1038/s41467-020-15886-6 (PMC7181746; doi:10.1038/s41467-020-15886-6)
Supplement: Supplementary file 2 — Description of Additional Supplementary Files [file 41467_2020_15886_MOESM2_ESM.docx]

**Description of Additional Supplementary Files**

File Name: Supplementary Data 1

Description: Clinical and sampling information of 10 CRC patients

File Name: Supplementary Data 2

Description: Tissue type and sequencing depth for 104 samples

File Name: Supplementary Data 3

Description: Somatic mutations for all tumor samples

File Name: Supplementary Data 4

Description: Potential driver genes

File Name: Supplementary Data 5

Description: Putative neoantigens predicted for 10 CRC patients

File Name: Supplementary Data 6

Description: Cancer cell fraction (CCF) values of mutations across all the samples
