# Supplementary material for: Mapping the spreading routes of lymphatic metastases in human colorectal cancer
Source: Nat Commun. 2020 Apr 24;11:1993. doi: 10.1038/s41467-020-15886-6 (PMC7181746; doi:10.1038/s41467-020-15886-6)
Supplement: Supplementary file 9 — Reporting Summary [file 41467_2020_15886_MOESM9_ESM.pdf]

nature research

Corresponding author(s): Fan Bai, Haizeng Zhang

Last updated by author(s): Mar 11, 2020

Reporting Summary

Nature Research wishes to improve the reproducibility of the work that we publish. This form provides structure for consistency and transparency in reporting. For further information on Nature Research policies, see Authors & Referees and the Editorial Policy Checklist .

Statistics

For all statistical analyses, confirm that the following items are present in the figure legend, table legend, main text, or Methods section.

n/a	Confirmed	
☐	☒	The exact sample size (n) for each experimental group/condition, given as a discrete number and unit of measurement
☐	☒	A statement on whether measurements were taken from distinct samples or whether the same sample was measured repeatedly
☐	☒	The statistical test(s) used AND whether they are one- or two-sided<br>*Only common tests should be described solely by name; describe more complex techniques in the Methods section.*
☐	☒	A description of all covariates tested
☐	☒	A description of any assumptions or corrections, such as tests of normality and adjustment for multiple comparisons
☐	☒	A full description of the statistical parameters including central tendency (e.g. means) or other basic estimates (e.g. regression coefficient) AND variation (e.g. standard deviation) or associated estimates of uncertainty (e.g. confidence intervals)
☐	☒	For null hypothesis testing, the test statistic (e.g. F, t, r) with confidence intervals, effect sizes, degrees of freedom and P value noted<br>*Give P values as exact values whenever suitable.*
☐	☒	For Bayesian analysis, information on the choice of priors and Markov chain Monte Carlo settings
☐	☒	For hierarchical and complex designs, identification of the appropriate level for tests and full reporting of outcomes
☐	☒	Estimates of effect sizes (e.g. Cohen's d, Pearson's r), indicating how they were calculated

Our web collection on statistics for biologists contains articles on many of the points above.

Software and code

Policy information about availability of computer code

Data collection	No special software was used.
Data analysis	BWA v0.5.9, Samtools v0.1.19, GATK v2.1-8, Picard tools v1.76, MuTect v1.1.4, Sequenza R package v.2.1.1, CNVkit v0.9.2, VarScan2 v2.3.9, POLYSOLVER v4, pVAC-seq v1.5.6, Pyclone v0.13.0, R v3.5.3, Python v3.5, NetMHCpan v4.0.

For manuscripts utilizing custom algorithms or software that are central to the research but not yet described in published literature, software must be made available to editors/reviewers. We strongly encourage code deposition in a community repository (e.g. GitHub). See the Nature Research guidelines for submitting code & software for further information.

Data

Policy information about availability of data

All manuscripts must include a data availability statement. This statement should provide the following information, where applicable:

- Accession codes, unique identifiers, or web links for publicly available datasets
- A list of figures that have associated raw data
- A description of any restrictions on data availability

The raw sequence data reported in this paper have been deposited in the Genome Sequence Archive in BIG Data Center, Beijing Institute of Genomics (BIG), Chinese Academy of Sciences, under accession numbers HRA000130 [https://bigd.big.ac.cn/gsa-human/s/A7hb3IW7] that are publicly accessible at https://bigd.big.ac.cn/gsa. The Genome Sequence Archive (GSA) is a data repository for archiving raw sequence reads. It accepts data submissions from all over the world and provides free access to all publicly available data for global scientific communities.

Field-specific reporting

Please select the one below that is the best fit for your research. If you are not sure, read the appropriate sections before making your selection.

- [x] Life sciences
- [ ] Behavioural & social sciences
- [ ] Ecological, evolutionary & environmental sciences

For a reference copy of the document with all sections, see nature.com/documents/nr-reporting-summary-flat.pdf

Life sciences study design

All studies must disclose on these points even when the disclosure is negative.

Sample size	No statistical methods were used to predetermine sample size. We retrieved high-quality exome sequencing data from 104 samples, including 28 primary tumors, 32 paracolic LNMs, 18 intermediate LNMs, 5 central LNMs, 11 LMs, and 10 matched normal tissues (Supplementary Data 1). The number of samples included was based on the number of samples available for analysis when the study was designed. All available samples that were suitable for inclusion were utilized. The number of samples analyzed was within the range of similar studies investigating the genetic relationship of primary tumors and paired metastases (Gerlinger et al., NEJM 2012; Xue, et al., Gastroenterology, 2016).
Data exclusions	There is no data that were excluded from the analyses.
Replication	There is no replication in our study.
Randomization	This was an observational study, no randomization was performed.
Blinding	Blinding was not considered appropriate for this study.

Reporting for specific materials, systems and methods

We require information from authors about some types of materials, experimental systems and methods used in many studies. Here, indicate whether each material, system or method listed is relevant to your study. If you are not sure if a list item applies to your research, read the appropriate section before selecting a response.

Materials & experimental systems

n/a	Involved in the study	
[x]	[ ]	Antibodies
[x]	[ ]	Eukaryotic cell lines
[x]	[ ]	Palaeontology
[x]	[ ]	Animals and other organisms
[ ]	[x]	Human research participants
[x]	[ ]	Clinical data

Methods

n/a	Involved in the study	
[x]	[ ]	ChIP-seq
[x]	[ ]	Flow cytometry
[x]	[ ]	MRI-based neuroimaging

Human research participants

Policy information about studies involving human research participants

Population characteristics	This study involves 10 treatment-naïve patients with microsatellite-stable (MSS) colorectal cancer.<br>Detailed clinical information of these patients were listed in Supplementary table 1.
Recruitment	We performed a prospective screen of colorectal cancer (CRC) patients who underwent radical resection in the Department of Colorectal Surgery, CHCAMS from October 2016 to October 2018. We applied a series of screening steps to select cases for this study: 1) 497 sporadic, microsatellite-stable (MSS) patients with informed consent were selected from 731 treatment-naïve patients; 2) 147 patients diagnosed with more than 2 LNMs were then selected; 3) all LNMs were classified into 3 subgroups: paracolic, intermediate, and central LNMs. A total of 29 patients with LNMs covering 2 or 3 subgroups of LNMs were selected (Patient_11 had only paracolic LNMs, but they were included due to the existence of liver metastasis); 4) 13 patients with samples that had sufficient and good-quality DNA were selected; and 5) 3 patients with low purity of the primary tumor were excluded (less than 20 somatic mutations detected). Finally, 10 CRC patients were selected. As only MSS CRC patients were selected, our results are restricted to these patients and may not apply to microsatellite instable (MSI) patients. Besides this, there are no other potential bias for recruitment in this dataset.
Ethics oversight	The present study was approved by the Institutional Review Board of Cancer Hospital, Chinese Academy of Medical Sciences (CHCAMS, Beijing, China).

Note that full information on the approval of the study protocol must also be provided in the manuscript.
